# Identification and prediction of m7G-related Alzheimer’s disease subtypes: insights from immune infiltration and machine learning models

**DOI:** 10.3389/fnagi.2023.1161068

**Published:** 2023-06-16

**Authors:** Chao Ma, Jian Li, Yuhua Chi, Xuan Sun, Maoquan Yang, Xueqin Sui

**Affiliations:** ^1^Department of General Medicine, Affiliated Hospital of Weifang Medical University, Weifang, Shandong, China; ^2^School of Clinical Medicine, Weifang Medical University, Weifang, Shandong, China; ^3^Department of Neurology, Affiliated Hospital of Weifang Medical University, Weifang, Shandong, China

**Keywords:** Alzheimer’s disease, m7G methylation, AD subtypes, immune infiltration, machine learning, predictive model

## Abstract

**Introduction:**

Alzheimer’s disease (AD) is a complex and progressive neurodegenerative disorder that primarily affects older individuals. N7-methylguanosine (m7G) is a common RNA chemical modification that impacts the development of numerous diseases. Thus, our work investigated m7G-related AD subtypes and established a predictive model.

**Methods:**

The datasets for AD patients, including GSE33000 and GSE44770, were obtained from the Gene Expression Omnibus (GEO) database, which were derived from the prefrontal cortex of the brain. We performed differential analysis of m7G regulators and examined the immune signatures differences between AD and matched-normal samples. Consensus clustering was employed to identify AD subtypes based on m7G-related differentially expressed genes (DEGs), and immune signatures were explored among different clusters. Furthermore, we developed four machine learning models based on the expression profiles of m7G-related DEGs and identified five important genes from the optimal model. We evaluated the predictive power of the 5-gene-based model using an external AD dataset (GSE44770).

**Results:**

A total of 15 genes related to m7G were found to be dysregulated in patients with AD compared to non-AD patients. This finding suggests that there are differences in immune characteristics between these two groups. Based on the differentially expressed m7G regulators, we categorized AD patients into two clusters and calculated the ESTIMATE score for each cluster. Cluster 2 exhibited a higher ImmuneScore than Cluster 1. We performed the receiver operating characteristic (ROC) analysis to compare the performance of four models, and we found that the Random Forest (RF) model had the highest AUC value of 1.000. Furthermore, we tested the predictive efficacy of a 5-gene-based RF model on an external AD dataset and obtained an AUC value of 0.968. The nomogram, calibration curve, and decision curve analysis (DCA) confirmed the accuracy of our model in predicting AD subtypes.

**Conclusion:**

The present study systematically examines the biological significance of m7G methylation modification in AD and investigates its association with immune infiltration characteristics. Furthermore, the study develops potential predictive models to assess the risk of m7G subtypes and the pathological outcomes of patients with AD, which can facilitate risk classification and clinical management of AD patients.

## Introduction

Alzheimer’s disease (AD) is a neurodegenerative disorder that affects the central nervous system and results in a progressive decline in cognitive function and memory. It predominantly affects the elderly population and significantly impacts patients’ quality of life (Cao and Zheng, 2018; Liu et al., 2020; Qiu et al., 2022). Recent statistics indicate that approximately 50 million individuals worldwide suffer from AD and other forms of dementia, and the number is increasing each year, resulting in a substantial burden on patients, families, society, and the healthcare system (Suresh et al., 2021). However, the pathogenesis of AD is exceedingly intricate, and its exact causative factors remain to be fully elucidated, impeding the development of AD drugs (Mangialasche et al., 2010; Ballard et al., 2011). Consequently, current treatments for AD are unsatisfactory. Nevertheless, recent advancements in bioinformatics, particularly leveraging the GEO database, have enabled the in-depth exploration of biomarkers that contribute to AD development, facilitating the development of multi-factor prediction models (Zhang T. et al., 2021; Li et al., 2022; Lin et al., 2022; Wang et al., 2022). These models can provide new insights into the individualized and precise treatment of AD patients.

There is mounting evidence to suggest that epigenetic modifications participate in the development of diseases by regulating gene expression post-transcriptionally (Zhang M. et al., 2021; Cheng et al., 2022; Luo et al., 2022). N7-methylguanosine (m7G) is a common RNA methylation that plays a crucial role in maintaining RNA processing, metabolism, stability, nuclear export, and translation (Wei et al., 2022; Zhong et al., 2022). Recent studies have shown a strong association between m7G and various pathological processes that affect the diagnosis and prognosis of diseases (Chen et al., 2022; Wei et al., 2022; Xia et al., 2023).

The role of the brain’s immune system, specifically the microglia, has been extensively investigated in the development of Alzheimer’s disease. Microglia, a specialized type of immune cell, play a crucial role in clearing debris and toxic substances from the brain (Jevtic et al., 2017). Identifying the precise role of microglia will aid researchers in developing more effective strategies to target this system, which could potentially prevent the disease from progressing in the early stages.

Several studies have indicated that m7G methylation is involved in the immune response to several diseases. RNA methylation in the immune system influences the maturation and response functions of immune cells (Lu et al., 2022). m7G modifications regulate innate immunity by influencing RNA immunogenicity and innate immune components in the body. Methylation modification patterns mediated by m7G regulators may be associated with tumor microenvironment infiltration in glioblastoma (Wu et al., 2022).

This study aimed to conduct a differential analysis of m7G regulators in AD and non-AD samples and examine the differences in immune features. Based on 15 differentially expressed m7G-related genes (M7RGs), we identified two m7G-related subtypes and assessed the differences in immune features between the subtypes. To investigate the biological processes involving m7G-related DEGs between the subtypes, we performed Gene Set Variation Analysis (GSVA). Additionally, we constructed four machine-learning models using m7G-related DEGs and evaluated the diagnostic power of multiple models using ROC curves.

## Materials and methods

### Data collection

Two datasets related to AD were collected from the GEO website database,^1^ namely GSE33000 and GSE44770. Both datasets contained samples obtained from human prefrontal cortex brain tissues. The GSE33000 dataset (GPL4372 platform) consisted of 157 healthy and 310 AD samples, which served as the training group. On the other hand, the GSE44770 dataset (GPL4372 platform) comprised 129 AD and 101 normal samples, and it was used as the test group. The gene expression profiles of the three datasets were normalized and processed using the “Perl” script and R package “limma.” Subsequently, 22 m7G regulators were identified from previous publications (Luo et al., 2022; Li et al., 2023; Maimaiti et al., 2023; Qin et al., 2023).

### Immune infiltration analysis

The study utilized the CIBERSORT algorithm to evaluate the relative abundances and infiltration scores of 22 immune cell types in AD samples, based on gene expression profiles (Gentles et al., 2015). To explore the relationship between M7RGs and immune cell types, correlation analysis was employed. A significance level of *P* < 0.05 was used. The results were presented using the R packages “reshape2” and “ggpubr.”

### Consensus clustering for AD patients

Based on the landscape of DEGs expression associated with AD, we classified AD samples into distinct m7G-related subtypes by utilizing the R package “Consensus Cluster Plus.” The maximum cluster number, *k* = 9 was selected, and the optimal cluster number was evaluated based on the consensus matrix (CM) and CDF. To evaluate the distribution between m7G-related clusters, we utilized Principal Component Analysis (PCA).

### GSVA

In this study, we employed GSVA, a differential analysis approach at the pathway level, to investigate the discrepancies in biological activities among the clusters of M7RGs. We implemented the GSVA method using the R package “GSVA.” We obtained GSVA gene sets from the “curated gene sets” and “ontology gene sets” modules available in the MSigDB database.

### Construction of machine-learning models and a nomogram

The cluster-specific DEGs were identified by intersecting the AD-related hub genes and the m7G cluster-related hub genes. Subsequently, the expression patterns of these DEGs were analyzed to construct four machine learning models using the R package “caret.” The models included the Random Forest (RF) (Rigatti, 2017), SVM (Gold and Sollich, 2003), Generalized Linear Model (GLM) (Nelder and Wedderburn, 1972), and eXtreme Gradient Boosting (XGB) (Romeo and Frontoni, 2022).

The study considered the distinct clusters as the response variable and selected the cluster-specific DEGs as explanatory variables. A total of 310 AD samples were randomly divided into a training set (*N* = 218, 70%) and a validation set (*N* = 92, 30%). The R package “caret” was used to control the training process with the parameter set to fivefold repeated cross-validation. Then, the “randomForest,” “kernlab,” and “xgboost” packages were loaded in sequence, and four machine learning algorithms, namely, RF, SVM, GLM, and XGB, were chosen. The “explain” function in the “DALEX” package was used to evaluate the performance of these models and generate various indicators related to the prediction results. The “DALEX” package was also utilized to visualize the residual distribution and feature importance among these models, while the “pROC” package was used to plot the area under the ROC curves. The top five predictive genes associated with AD were identified based on the optimal machine learning model. Finally, the diagnostic value of the model was verified using the ROC curve analysis in the GSE44770 dataset.

We have developed a nomogram to predict the risk of AD patients by utilizing the five essential genes identified from the RF model. To assess the predictive performance of the nomogram, we utilized decision curve analysis and a calibration curve. These methods were employed to validate the efficacy of the predictive model.

### Independent validation analysis

We opted to use the AD dataset GSE44770 to assess the diagnostic efficiency of a 5-gene-based RF model. We presented the outcomes in the form of ROC curves. Additionally, we developed a nomogram employing the GSE44770 dataset to assess the risk of m7G subtypes. The calibration curve and DCA were employed to evaluate the predictive performance of the nomogram model.

### Statistical analysis

The statistical analysis was performed using R software (Version 4.2.1), with the Perl and “limma” packages used for data processing. The sample classification was carried out using the “Consensus Cluster Plus” package. For continuous variables, normality was assessed, and either the Student’s *t*-test or Wilcoxon rank-sum test was employed for analysis. The Chi-square test was used for categorical variable differences. A two-sided adjusted *p*-value of < 0.05 was considered statistically significant.

## Results

### Identification of differentially expressed M7RGs

**FIGURE 1:**
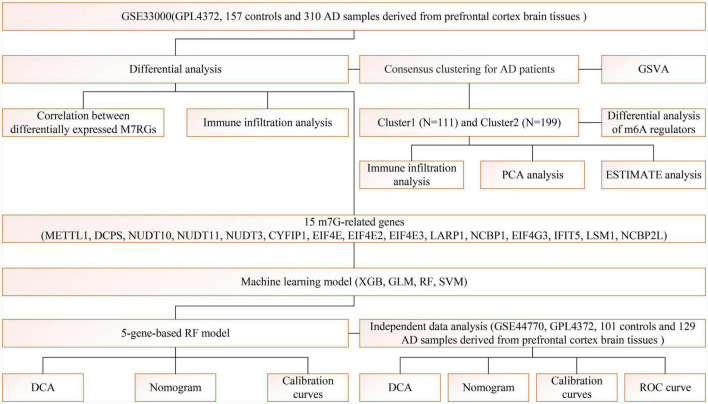
The study flow chart.

**FIGURE 2:**
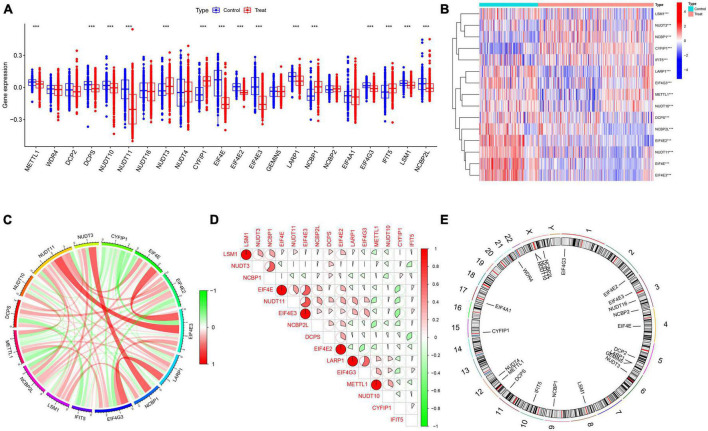
Identification of differentially expressed m7G-related genes. **(A)** Boxplots showed the expression of 22 M7RGs between AD and non-AD controls ***p < 0.001. **(B)** The expression patterns of 15 M7RGs were presented in the heatmap. **(C)** Interactions between 15 differentially expressed M7RGs at the molecular level. **(D)** Correlation analysis of 15 differentially expressed M7RGs. Red and green colors represent positive and negative correlations, respectively. The correlation coefficients were marked with the area of the pie chart. **(E)** The location of 22 M7RGs on chromosomes.

A detailed flow chart of the study process is exhibited in Figure 1. In order to investigate the biological significance of m7G in AD, we examined the expression profiles of 22 M7RGs and performed differential analysis on AD and normal samples using the GSE33000 dataset. From this analysis, we identified 15 differentially expressed M7RGs, with 4 of them (NUDT3, CYFIP1, NCBP1, and IFIT5) being upregulated in AD patients, while 11 of them (METTL1, DCPS, NUDT10, NUDT11, EIF4E, EIF4E2, EIF4E3, LARP1, EIF4G3, LSM1, and NCBP2L) were downregulated compared to non-AD patients (Figures 2A, B, E). In addition, we conducted a correlation analysis to explore the relationship between these differentially expressed M7RGs. Our results indicated that EIF4E3 was significantly positively correlated with NUDT11 and negatively correlated with CYFIP1 (Figures 2C, D).

### Immune landscape analysis

**FIGURE 3:**
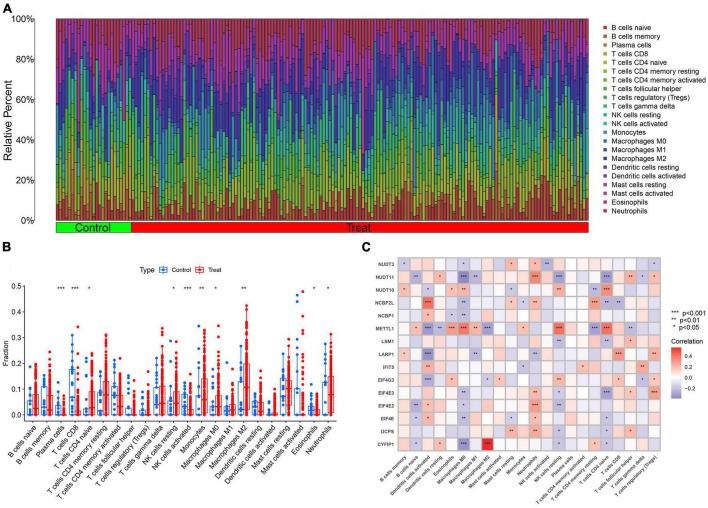
The analysis of immune features between AD and non-AD samples. **(A)** The relative abundances of 22 infiltrated immune cells between AD and non-AD controls. **(B)** Boxplots showed the differences in immune infiltration between AD and non-AD controls. **(C)** Correlation analysis between 15 M7RGs and 22 immune cell types. *p < 0.05, **p < 0.01, ***p < 0.001.

The CIBERSORT analysis was utilized to assess the differences in immune features between AD and normal samples (Figure 3A). The results of our study revealed that AD samples had elevated levels of infiltrating naive CD4^+^ T cells, resting NK cells, monocytes, M0, M2 macrophages, and neutrophils, whereas infiltration levels of plasma cells, CD8^+^ T cells, activated NK cells, and eosinophils were decreased (Figure 3B). These findings suggest that alterations in the immune microenvironment play a crucial role in the development of AD. Moreover, the correlation analysis demonstrated that M2 macrophages exhibited a positive correlation with CYFIP1 and a negative correlation with METTL1. In contrast, neutrophils showed a positive association with the M7RGs (excluding LARP1), while M0 macrophages and naive CD4^+^ T cells were negatively associated with the M7RGs (excluding METTL1 and NUDT1) (Figure 3C). These results suggest that M7RGs may participate in the onset and progression of AD by affecting immune cell infiltration levels. Moreover, the results of the CIBERSORTx analysis are shown in Supplementary Figure 1 and Supplementary Table 1.

### Identification of m7G clusters in AD

**FIGURE 4:**
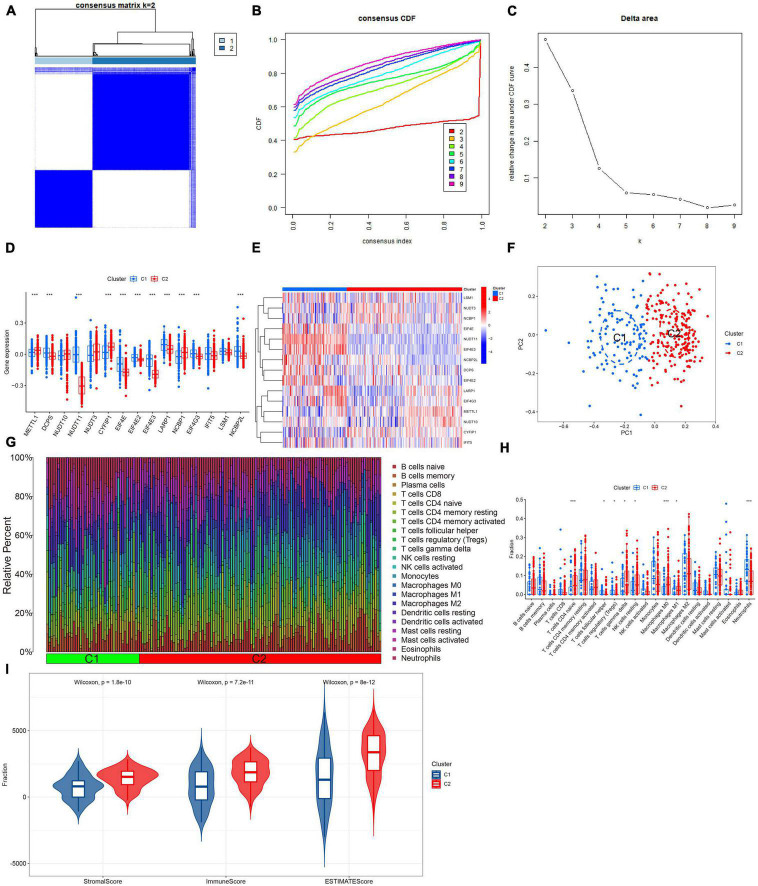
Identification of m7G-related molecular subtypes in AD. Consensus clustering matrix when *k* = 2 **(A)**, representative cumulative distribution function (CDF) curves **(B)**, CDF delta area curves **(C)**. **(D)** Boxplots showed the expression of 15 M7RGs between two m7G clusters ***p < 0.001. **(E)** The different analyses of 15 M7RGs between two m7G clusters were presented in the heatmap. **(F)** PCA analysis for cluster 1 and cluster 2. **(G)** The relative abundances of 22 infiltrated immune cells between two m7G clusters. **(H)** Boxplots showed the differences in immune infiltration between two m7G clusters *p < 0.05, ***p < 0.001. **(I)** Violin plots showed the StromalScore, ImmuneScore, and ESTIMATEScore between two m7G clusters.

Based on the differential expression of M7RGs, we conducted a consensus clustering analysis to investigate new molecular subtypes for patients with AD. The consensus clustering algorithm classified AD patients into two m7G-related subtypes when K was equal to 2 (Figures 4A–C). The PCA analysis revealed that the two clusters showed distinct molecular mechanisms related to m7G (Figure 4F).

### Differential analysis of m7G regulators and immune signatures between m7G clusters

In this study, we performed a differential analysis of 15 M7RGs to investigate molecular signatures between two subtypes, Cluster 1 and Cluster 2. Our results showed that Cluster 1 exhibited elevated expression levels of DCPS, NUDT11, EIF4E, EIF4E2, EIF4E3, LARP1, EIF4G3, and NCBP2L, while Cluster 2 showed elevated expression levels of METTL1, CYFIP1, and NCBP1, as depicted in Figures 4D, E. Moreover, based on the CIBERSORT results, we observed a significant difference in immune cell infiltration between the two m7G-related subtypes, with Cluster 1 showing higher levels of regulatory T cells (Tregs) and neutrophils and Cluster 2 showing higher levels of naive CD4^+^ T cells, resting NK cells, M0, and M1 macrophages (Figure 4H). We also conducted ESTIMATE analysis, which revealed that Cluster 2 had a higher StromalScore, ImmuneScore, and ESTIMATEScore, indicating elevated immune infiltration levels (Figures 4G, I).

### Enrichment analysis

**FIGURE 5:**
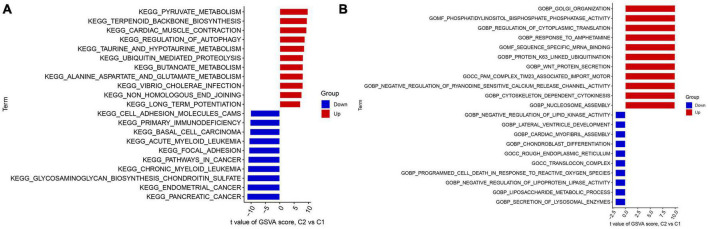
Identification of biological activities between two m7G clusters. **(A)** The KEGG pathway analysis. **(B)** The GO function analysis.

The study utilized GSVA analysis to investigate the differences in biological activities between two AD subtypes. The results showed that Cluster 1 had reinforced cell adhesion molecules, primary immunodeficiency, focal adhesion, and cancer-related pathways. In contrast, Cluster 2 showed activation of terpenoid backbone biosynthesis, regulation of autophagy, ubiquitin-mediated proteolysis, vibrio cholerae infection, and metabolism-related pathways (Figure 5A). Furthermore, functional enrichment analysis revealed that Cluster 1 was significantly positively correlated with the secretion of lysosomal enzymes, the liposaccharide metabolic process, the translocon complex, the negative regulation of lipoprotein lipase activity, and lipid kinase activity. Conversely, Cluster 2 showed enrichment in golgi organization, nucleosome assembly, Wnt protein secretion, response to amphetamine, and regulation of cytoplasmic translation (Figure 5B). Thus, it was inferred that Cluster 2 may be involved in mRNA processing, translation, and metabolic pathways.

### Construction of machine-learning models

**FIGURE 6:**
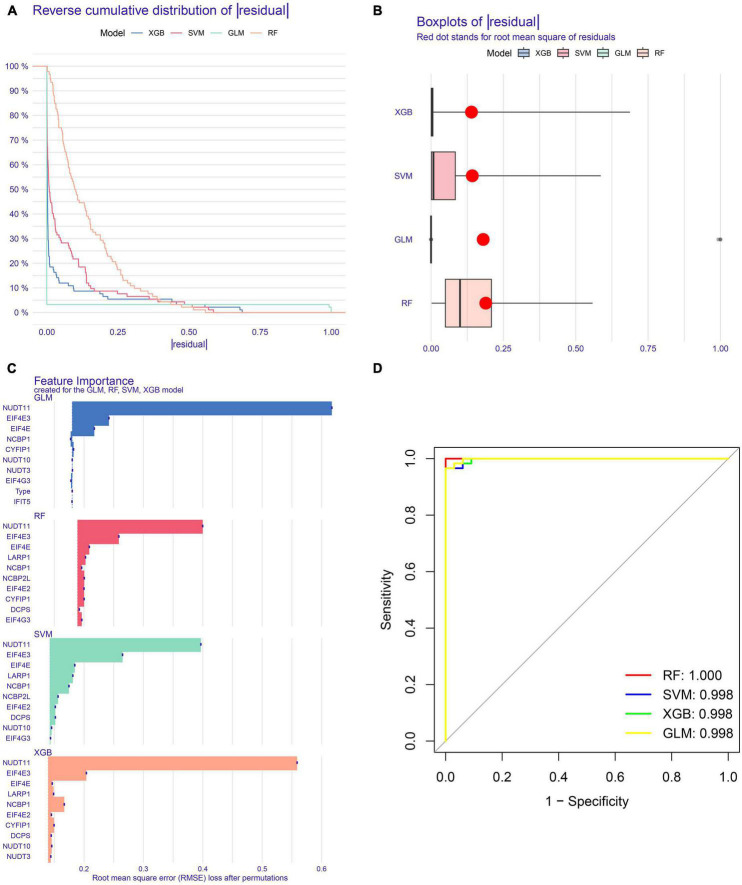
Construction and evaluation of RF, SVM, GLM, and XGB machine models. **(A)** Cumulative residual distribution of each machine learning model. **(B)** Boxplots showed the residuals of each machine learning model. Red dot represented the root mean square of residuals (RMSE). **(C)** The important features in RF, SVM, GLM, and XGB machine models. **(D)** ROC curves of four machine learning models based on fivefold repeated cross-validation in the testing cohort.

To explore the m7G regulators associated with AD subtypes, we constructed four machine-learning models, including RF, SVM, GLM, and XGB using the 15 m7G-related DEGs in the AD training set. The XGB and GLM models showed lower residuals (Figures 6A, B). We identified 10 key genes from four modules, ranked by root mean square error (RMSE) (Figure 6C). In addition, we used the ROC curve to evaluate the diagnostic efficacy of the four models. The RF model exhibited the strongest diagnostic power (AUC = 1.000) (Figure 6D). Finally, we selected the top five best factors (NCBP2L, LARP1, EIF4E, EIF4E3, and NUDT11) from the RF model as predictor genes for further analysis.

**FIGURE 7:**
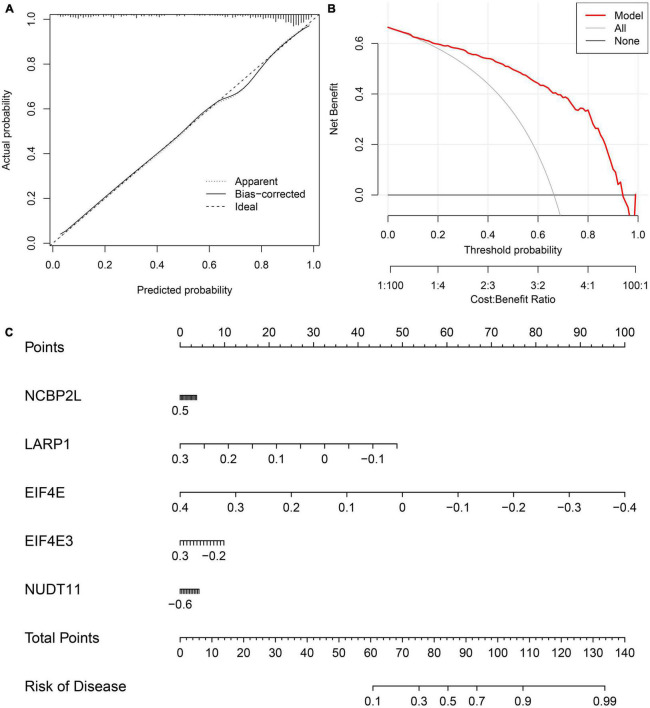
Validation of the 5-gene-based RF model. Construction of the calibration curve **(A)** and DCA **(B)** for assessing the predictive efficiency of the nomogram model. **(C)** Construction of a nomogram for predicting the risk of AD clusters based on the 5-gene-based RF model.

**FIGURE 8:**
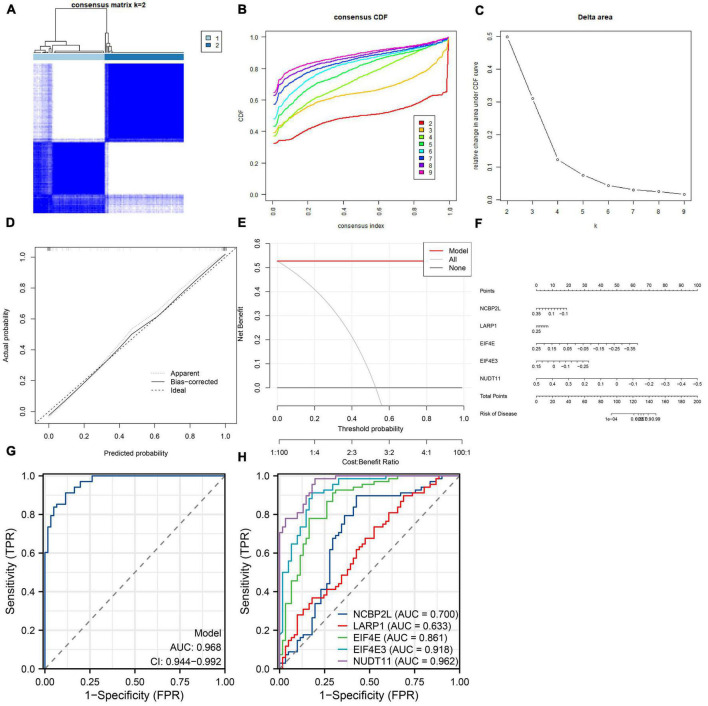
Independent validation analysis based on the GSE44770 dataset. Consensus clustering matrix when *k* = 2 **(A)**, representative cumulative distribution function (CDF) curves **(B)**, CDF delta area curves **(C)**. Construction of the calibration curve **(D)** and DCA **(E)** for assessing the predictive efficiency of the nomogram model. **(F)** Construction of a nomogram for predicting the risk of AD clusters based on the 5-gene-based RF model. **(G)** ROC curve of the 5-gene-based RF model. **(H)** ROC curves of NCBP2L, LARP1, EIF4E, EIF4E3, NUDT11.

We developed a nomogram to assess the predictive ability of the RF model across various AD subtypes (Figure 7C). To validate the predictive performance of the nomogram, we employed a calibration curve and DCA. The calibration curve showed that the predicted risk of AD clusters was nearly identical to the actual risk (Figure 7A). Furthermore, the DCA result demonstrated that the nomogram had a superior predictive ability, providing a theoretical basis for the prediction of m7G-related AD subtypes (Figure 7B). Subsequently, we classified AD patients into two m7G subtypes using a consensus clustering algorithm based on the GSE44770 dataset (Figures 8A–C). A nomogram was conducted to assess the risk of AD subtypes (Figure 8F). The calibration curve and DCA analysis were used to evaluate the diagnostic capability of the nomogram model (Figures 8D, E). The ROC analysis revealed an AUC of 0.968 for the 5 gene-based RF model in the GSE44770 dataset, demonstrating the higher diagnostic efficacy of our predictive model for AD subtypes (Figure 8G). Furthermore, the AUC values for NCBP2L, LARP1, EIF4E, EIF4E3, and NUDT11 were 0.700, 0.633, 0.861, 0.918, and 0.962, respectively (Figure 8H).

## Discussion

The etiology of AD is complex, and its diagnosis is challenging. Consequently, AD patients often receive inadequate treatment. The traditional classification of AD based on histology poses a significant challenge to drug treatment due to drug resistance. Therefore, there is an urgent need to develop effective predictive models and molecular subtypes for risk stratification and personalized treatment of AD patients.

RNA methylation post-transcriptionally regulates target RNA metabolism and function, either promoting or inhibiting disease development (Dai et al., 2021; Cui et al., 2022). One of the most common chemical modifications is m7G, installed by key methyltransferases such as METTL1/WDR4, RAM/RNMT, and WBSCR22/TRMT112 (Luo et al., 2022). METTL1 has been identified as a high-risk gene in hepatocellular carcinoma (LIHC), contributing to tumor-associated phenotypes by inhibiting PTEN signaling (Tian et al., 2019). WDR4 plays a crucial role in promoting the proliferation of hepatocellular carcinoma by mediating m7G methylation (Xia et al., 2021). In pancreatic cancer (PAAD), the WBSCR22/TRMT112 complex downregulates the oncogene ISG15 expression, impairing malignant phenotypes (Khan et al., 2022).

The immune system is recognized as a crucial factor in AD, with various components of the immune system in both the brain and periphery interacting to contribute to the disease. The classical immune components of the nervous system, such as microglia and complement, as well as novel aspects of the peripheral immune system, such as monocytes and lymphocytes, are all implicated in AD.

Lu et al. (2022) found that most M7RGs are highly expressed in COVID-19 patients compared with that in non-COVID-19 patients. And m7G-cluster B showed higher immune infiltration and milder symptoms. Furthermore, m7G modification has been shown to play an essential role in the development of cardiovascular disease. Wei et al. (2022) indicated that m7G methylation is involved in the progression of pulmonary arterial hypertension (PAH) by affecting the immune microenvironment. However, the role of m7G in AD and its relationship with immunity remain incompletely understood.

Our objective was to investigate the biological activities of M7RGs in AD progression and identify m7G-related clusters and immune signatures. Additionally, we aimed to construct an optimal machine-learning model for evaluating the risk of AD subtypes. In this study, we obtained the expression profile of M7RGs from the GSE33000 dataset and performed differential analysis between AD and non-AD individuals. Our data revealed dysregulation of 15 M7RGs in AD patients. A correlation analysis between m7G regulators was conducted to unveil the relationship between m7G regulators and AD. Furthermore, CIBERSORT analysis showed an immune infiltration difference between AD and non-AD patients. Infiltration levels of naive CD4^+^ T cells, resting NK cells, neutrophils, and M0 and M2 macrophages were found to be elevated in AD patients.

The results from GSVA indicated that Cluster 1 was mainly enriched in cell adhesion molecules, primary immunodeficiency, focal adhesion, and cancer-related pathways. On the other hand, Cluster 2 was characterized by metabolism-related pathways, mRNA processing, and translation. Furthermore, Cluster 2 was enriched in terpenoid backbone biosynthesis, regulation of autophagy, ubiquitin-mediated proteolysis, vibrio cholerae infection, and metabolism-related pathways (Figure 7B). Additionally, functional enrichment analyses indicated that Cluster 1 was strongly associated with the secretion of lysosomal enzymes, the liposaccharide metabolic process, the translocon complex, the negative regulation of lipoprotein lipase activity, and lipid kinase activity. However, Cluster 2 was enriched in golgi organization, nucleosome assembly, Wnt protein secretion, the response to amphetamine, and the regulation of cytoplasmic translation (Figure 7C). Thus, we postulate that Cluster 2 may play a role in regulating amino acid metabolism-related pathways, mRNA processing, and translation. Notably, previous studies have reported that taurine and glutamate intake could improve learning and memory function and delay the progression of Alzheimer’s disease. Consistent with these findings, Cluster 2 showed a stronger activity of taurine- and glutamate-related metabolism pathways. Taken together, these results suggest that Cluster 2 may have more activated amino acid metabolism-related pathways to halt the development of Alzheimer’s disease.

Mining hub genes with higher diagnostic value for a disease using multiple machine learning models is becoming a common research method. In this study, we constructed four machine-learning models to screen five important genes associated with AD from 15 m7G-related DEGs. We used ROC curves to assess the predictive power of the four models, and the results suggested that the RF model exhibited relatively higher diagnostic performance (AUC = 1.000) and has the potential to distinguish between different AD subgroups. We established a nomogram to assess the risk of AD subtypes based on the expression profiles of five important genes, including NCBP2L, EIF4E, EIF4E3, LARP1, and NUDT11. The DCA and calibration curve further indicated that this model exhibited relatively higher diagnostic value and potential for clinical application.

Kenny et al. (2019) reported that EIF4E could serve as a specific biomarker for diagnosing AD. The phosphorylated levels of EIF4E were found to be significantly higher in patients with advanced AD, which correlated positively with the phosphorylated Tau protein levels. Therefore, the phosphorylated EIF4E protein might promote the progression of AD by enhancing the accumulation of phosphorylated Tau protein in neurons (Li et al., 2004). Furthermore, the study revealed that the level of differentially methylated regions (DMRs) was associated with various dementia-related genes, including EIF4E, that contribute to the onset of AD (Pérez et al., 2022).

To further validate the diagnostic efficacy of the 5-gene-based RF model, we selected an external AD dataset (GSE44770). The AUC value is 0.968 in GSE44770, suggesting that the model has potential for a wide range of applications in AD diagnosis.

## Conclusion

Our study revealed that dysregulated m7G regulators are commonly found in patients with AD, which have an impact on the immune microenvironment. We identified two clusters of m7G and analyzed the differences in immune features between these two clusters. We chose a 5-gene-based RF model as the optimal machine learning model, which can accurately assess the risk of different subtypes of AD. Our findings elucidate the biological significance of m7G regulators in AD and provide a valuable insight for the risk stratification and clinical treatment of AD.

## Data availability statement

The data presented in this study are deposited in the Gene Expression Omnibus (GEO, http://www.ncbi.nlm.nih.gov/geo/) repository, accession numbers: GSE33000 and GSE44770.

## Author contributions

XS designed the study, reviewed, and edited the manuscript. CM and JL drafted the manuscript. YC, XS, and MY performed the bioinformatic analysis. All authors contributed to the article and approved the submitted version.

## Footnotes

1
www.ncbi.nlm.nih.gov/geo
