# Supplementary material for: Identification and prediction of m7G-related Alzheimer’s disease subtypes: insights from immune infiltration and machine learning models
Source: Front Aging Neurosci. 2023 Jun 16;15:1161068. doi: 10.3389/fnagi.2023.1161068 (PMC10312082; doi:10.3389/fnagi.2023.1161068)
Supplement: Supplementary file 1 [file Data_Sheet_1.ZIP › Supplementary material/Supplementary Figure 1.pdf]

CIBERSORTx Output Display

Input Sample	B cells naive	B cells memory	Plasma cells	T cells CD8	T cells CD4 naive	T cells CD4 memory resting	T cells CD4 memory activated	T cells follicular helper	T cells regulatory (Tregs)	T cells gamma delta	NK cells resting	NK cells activated	Monocytes	Macrophages M0	Macrophages M1	Macrophages M2	Dendritic cells resting	Dendritic cells activated	Mast cells resting	Mast cells activated	Eosinophils	Neutrophils	P-value	Correlation
GSM1424090_Control	0.078	0	0	0	0	0.087	0.021	0	0.089	0	0.003	0.037	0.257	0	0	0	0	0.008	0.176	0	0.043	0.200	0.000	0.339
GSM1424091_Control	0	0.047	0.11	0.308	0.181	0	0.031	0.025	0	0	0	0.081	0	0.044	0	0	0	0	0.174	0	0	0.000	0.000	-0.016
GSM1424092_Control	0	0.056	0.038	0.118	0	0.094	0.041	0	0.018	0	0.061	0	0.023	0	0	0.184	0.046	0	0	0.261	0.061	0.000	0.000	0.226
GSM1424093_Control	0.091	0	0	0.201	0.046	0	0.072	0.035	0.04	0	0	0.109	0	0	0	0	0	0.063	0.269	0	0.073	0.000	0.000	-0.054
GSM1424094_Control	0	0.059	0.063	0.438	0	0	0.159	0	0	0	0.066	0.018	0	0	0.056	0	0.035	0	0.049	0.058	0	0.000	0.000	0.111
GSM1424095_Control	0.104	0	0	0.033	0.024	0	0	0	0.043	0	0.041	0.036	0.203	0	0.033	0.175	0.055	0	0.136	0	0	0.117	0.000	0.270
GSM1424096_Control	0	0.039	0.15	0.054	0.207	0	0.104	0.081	0	0	0	0.175	0	0.032	0	0	0.003	0	0.155	0	0	0.000	0.000	0.025
GSM1424097_Control	0	0.051	0.022	0.25	0.109	0	0.158	0	0.046	0	0	0	0	0.055	0	0	0.059	0	0.25	0	0	0.000	0.000	-0.018
GSM1424098_Control	0	0.161	0.014	0	0.068	0	0	0	0.209	0	0.113	0	0.077	0.078	0.026	0	0	0	0.128	0	0	0.126	0.000	0.022
GSM1424099_Control	0.056	0	0.174	0.112	0	0	0.092	0	0.17	0	0.029	0	0	0.114	0	0	0.089	0.069	0.095	0	0	0.000	0.000	-0.055
GSM1424100_Control	0.013	0.109	0	0.013	0	0.169	0.108	0.144	0	0	0	0.006	0	0	0.203	0.137	0	0	0.1	0	0	0.000	0.000	0.326
GSM1424101_Control	0	0.068	0.095	0.245	0	0	0.231	0	0.047	0	0	0.107	0	0.064	0	0	0	0	0.131	0	0.013	0.000	0.000	-0.000
GSM1424102_Control	0	0.048	0.011	0.253	0	0	0.167	0	0.02	0	0	0.106	0	0.063	0	0.029	0.003	0	0.191	0	0.109	0.000	0.000	0.084
GSM1424103_Control	0	0.075	0.034	0.125	0.136	0	0.215	0	0	0.054	0	0.1	0	0	0	0	0.027	0	0.184	0	0.051	0.000	0.000	0.110
GSM1424104_Control	0	0.084	0.083	0	0.045	0.097	0.126	0.189	0	0	0.176	0	0	0.096	0.031	0	0.008	0	0.064	0	0	0.000	0.000	0.025
GSM1424105_Control	0.156	0	0	0.141	0	0.196	0.059	0.012	0	0	0.097	0.014	0	0	0.04	0	0	0	0.15	0	0.137	0.000	0.000	0.067
GSM1424106_Control	0.083	0	0.036	0.226	0.027	0	0.241	0	0	0	0	0.088	0	0.156	0	0	0	0	0.143	0	0	0.000	0.000	0.041
GSM1424107_Control	0	0.024	0.012	0.399	0	0	0.054	0.037	0.03	0	0	0.103	0	0.117	0	0	0	0	0.126	0	0.098	0.000	0.000	0.090
GSM1424108_Control	0	0.075	0.062	0.318	0.122	0	0	0	0.075	0	0	0.133	0	0.122	0	0	0	0	0.094	0	0	0.000	0.000	-0.033
GSM1424109_Control	0.167	0	0	0	0.134	0.055	0.078	0	0.012	0	0.054	0.127	0	0.068	0	0.055	0.046	0	0.205	0	0	0.000	0.000	0.070
GSM1424110_Control	0	0.054	0.01	0.273	0.013	0	0.11	0	0.065	0.221	0	0	0	0.019	0.005	0	0.034	0	0	0.169	0.027	0.000	0.000	0.218
GSM1424111_Control	0	0.147	0.065	0.235	0.073	0	0.113	0	0	0	0	0.107	0	0	0.006	0	0.053	0	0.131	0	0.07	0.000	0.000	0.009
GSM1424112_Control	0	0.106	0.089	0.351	0.062	0	0.179	0.036	0	0	0	0.037	0	0.001	0	0	0.02	0.014	0.105	0	0	0.000	0.000	0.085
GSM1424113_Control	0	0.121	0	0.082	0.177	0	0	0.07	0	0.007	0	0.035	0	0	0.033	0.116	0.059	0	0.221	0	0.047	0.031	0.000	0.032
GSM1424114_Control	0	0.082	0.065	0.287	0	0	0	0.126	0	0	0	0.068	0	0.036	0.046	0.138	0	0.013	0.128	0	0.01	0.000	0.000	0.048
GSM1424115_Control	0	0.147	0	0.184	0.059	0	0.098	0.186	0	0	0	0.077	0	0.091	0.015	0	0	0	0.118	0	0.025	0.000	0.000	0.046
GSM1424116_Control	0	0.083	0.146	0.098	0	0.041	0.075	0	0.12	0	0	0.136	0	0.019	0	0	0.018	0	0.265	0	0	0.000	0.000	0.094
GSM1424117_Control	0	0.127	0.126	0.169	0	0.089	0.068	0	0	0	0	0.125	0	0	0	0.143	0.079	0	0.072	0	0	0.000	0.000	0.147
GSM1424118_Control	0	0.013	0.043	0.104	0	0.139	0.302	0	0.016	0	0.139	0	0	0.097	0.011	0	0	0	0.135	0	0	0.000	0.000	0.022
GSM1424119_Control	0.111	0	0	0	0	0.139	0.154	0	0.113	0.253	0.057	0	0	0.035	0.009	0	0.069	0	0.047	0	0	0.014	0.000	0.044
GSM1424120_Control	0	0.096	0.054	0.262	0	0	0.078	0.04	0	0	0.072	0.076	0	0	0	0	0.07	0	0.198	0	0.052	0.000	0.000	0.071
GSM1424121_Control	0.056	0	0.103	0	0.101	0	0.186	0.15	0	0.088	0	0.033	0	0.111	0	0	0	0	0.172	0	0	0.000	0.000	-0.012
GSM1424122_Control	0	0.114	0.129	0.141	0.039	0	0.267	0.013	0	0.015	0	0.037	0	0.061	0	0	0.017	0	0.169	0	0	0.000	0.000	0.016
GSM1424123_Control	0.093	0	0	0.308	0	0	0.097	0	0.128	0	0.081	0	0	0.186	0	0	0	0	0.079	0	0.028	0.000	0.000	-0.013
GSM1424124_Control	0	0.159	0.216	0.176	0	0.076	0.33	0	0.014	0	0	0	0	0.002	0	0	0	0	0.027	0	0	0.000	0.000	0.021
GSM1424125_Control	0	0.076	0.066	0.159	0	0	0.145	0.007	0.125	0	0	0.062	0	0.053	0	0	0.01	0	0.28	0	0.017	0.000	0.000	-0.005
GSM1424126_Control	0	0.203	0.078	0.196	0	0	0.102	0.005	0	0	0	0	0	0	0.028	0.055	0.042	0.014	0.213	0	0.065	0.000	0.000	-0.004
GSM1424127_Control	0	0	0.187	0.216	0.145	0	0.088	0.061	0.031	0.09	0	0.056	0	0	0.035	0	0	0	0.09	0	0	0.000	0.000	0.092
GSM1424128_Control	0	0.016	0.053	0.35	0	0	0.135	0.064	0	0	0	0.066	0	0	0.007	0	0	0	0.224	0	0.084	0.000	0.000	0.049
GSM1424129_Control	0	0.045	0.095	0	0.336	0	0	0	0.147	0.054	0.065	0	0	0	0	0	0.069	0	0.018	0.098	0	0.072	0.000	0.015
GSM1424130_Control	0	0.094	0.11	0.297	0	0	0.213	0.023	0	0	0	0.046	0	0.026	0	0	0.035	0	0.157	0	0	0.000	0.000	-0.032
GSM1424131_Control	0.108	0	0.026	0.098	0.038	0	0.061	0	0	0	0.071	0.118	0	0	0	0	0.089	0	0.267	0	0.124	0.000	0.000	-0.018
GSM1424132_Control	0.05	0	0.016	0.174	0.103	0	0.109	0.043	0	0	0	0.156	0	0.039	0	0	0.012	0	0.2	0	0.098	0.000	0.000	0.003
GSM1424133_Control	0	0.161	0.162	0	0	0.34	0.016	0	0.022	0	0.086	0.002	0	0.089	0	0	0	0	0.002	0	0.119	0.000	0.000	0.031
GSM1424134_Control	0	0.154	0.08	0.149	0	0.002	0.059	0	0.119	0	0.048	0.127	0	0.087	0.017	0	0	0	0.114	0	0.044	0.000	0.000	0.099
GSM1424135_Control	0	0.199	0.042	0.12	0.225	0	0.02	0	0.095	0	0	0.088	0	0.11	0.005	0	0.087	0	0	0.009	0	0.000	0.000	0.100
GSM1424136_Control	0.115	0	0.01	0.221	0.031	0	0	0.078	0	0	0	0.145	0	0	0	0	0.059	0	0.298	0	0.043	0.000	0.000	-0.057
GSM1424137_Control	0	0.01	0.095	0.26	0	0	0.166	0.039	0	0	0	0.095	0	0	0	0	0	0	0.143	0	0.193	0.000	0.000	-0.017
GSM1424138_Control	0	0.062	0.087	0	0.166	0.153	0.075	0	0	0.009	0.121	0	0	0.222	0.004	0	0	0	0.096	0	0.006	0.000	0.000	0.079
GSM1424139_Control	0	0.104	0.132	0.452	0	0	0.111	0	0	0.03	0	0	0	0.058	0	0	0	0	0.098	0	0.015	0.000	0.000	0.058
GSM1424140_Control	0	0.075	0.125	0.215	0	0	0.068	0.307	0	0.013	0	0.029	0	0.019	0	0	0.024	0.025	0.099	0	0	0.000	0.000	-0.027
GSM1424141_Control	0.063	0	0	0.233	0	0.076	0.112	0	0.048	0	0	0.002	0	0.216	0.035	0	0	0	0.181	0	0.033	0.000	0.000	0.053
GSM1424142_Control	0.085	0	0	0.174	0	0.053	0.141	0.071	0	0	0.036	0.044	0	0.128	0.029	0	0.029	0	0.18	0	0.03	0.000	0.000	0.024
GSM1424143_Control	0	0	0.113	0.113	0	0.078	0.144	0	0	0	0.079	0	0	0.086	0.032	0	0.097	0	0.181	0	0.077	0.000	0.000	-0.087
GSM1424144_Control	0	0.187	0.101	0.312	0.022	0	0.088	0.063	0.04	0	0.003	0.104	0	0.03	0	0	0.008	0	0.032	0	0.01	0.000	0.000	0.140
GSM1424145_Control	0.092	0	0	0	0.009	0	0.045	0	0.25	0	0	0.121	0	0.116	0.04	0	0.017	0	0.33	0	0	0.000	0.000	0.004
GSM1424146_Control	0	0.105	0.073	0.162	0.064	0	0.118	0.116	0	0.076	0	0.049	0	0.09	0	0	0.004	0	0.143	0	0	0.000	0.000	-0.000
GSM1424147_Control	0	0.08	0.1	0.363	0	0	0.111	0	0.183	0	0	0.074	0	0	0.031	0	0.005	0	0	0.035	0.016	0.000	0.000	0.092
GSM1424148_Control	0	0.029	0.012	0.246	0	0	0.111	0.101	0	0.194	0	0.05	0	0.033	0	0	0.054	0	0.151	0	0.019	0.000	0.000	0.204
GSM1424149_Control	0	0.145	0.08	0.013	0	0.026	0.205	0.066	0	0.167	0	0.105	0	0.017	0.03	0	0	0	0.129	0	0.017	0.000	0.000	0.040
GSM1424150_Control	0	0	0.148	0.137	0	0	0.184	0.01	0.072	0.151	0	0.197	0	0	0	0	0	0	0.101	0	0	0.000	0.000	0.061
GSM1424151_Control	0	0.033	0.114	0.167	0	0.033	0.115	0.168	0	0.082	0	0.043	0	0.032	0.039	0	0.078	0	0.096	0	0	0.000	0.000	0.046
GSM1424152_Control	0	0	0.025	0.025	0	0.083	0	0.084	0	0	0	0.072	0.074	0	0.071	0	0.003	0.051	0	0.44	0.024	0.048	0.000	0.540
GSM1424153_Control	0	0	0.112	0.29	0	0	0.219	0	0.057	0	0.037	0.043	0	0.006	0	0	0	0.016	0.144	0	0.076	0.000	0.000	0.021
GSM1424154_Control	0	0.132	0	0.066	0.336	0	0	0	0	0.062	0	0.011	0	0.201	0	0.007	0.015	0	0.101	0	0.071	0.000	0.000	-0.005
GSM1424155_Control	0	0.117	0.048	0.177	0.06	0	0.196	0	0	0.125	0	0.08	0	0	0	0	0.01	0	0.154	0	0.034	0.000	0.000	0.205
GSM1424156_Control	0	0	0.069	0.43	0	0	0.084	0.085	0	0	0	0.113	0	0.004	0.037	0	0.061	0	0.117	0	0	0.000	0.000	0.135
GSM1424157_Control	0.131	0	0	0.168	0.11	0	0.005	0.009	0.035	0	0	0.14	0	0.075	0	0	0	0.017	0.288	0	0.021	0.000	0.000	-0.000
GSM1424158_Control	0	0.046	0.008	0.259	0	0	0.125	0.02	0.034	0	0	0.161	0	0	0	0	0.009	0	0.269	0	0.07	0.000	0.000	-0.013
GSM1424159_Control	0	0.116	0.055	0.317	0.111	0	0.18	0	0	0	0	0.005	0	0.073	0	0	0	0	0.142	0	0	0.000	0.000	0.005
GSM1424160_Control	0	0.133	0.232	0.024	0	0.065	0	0	0.059	0	0.098	0.103	0	0.152	0	0	0.009	0	0.125	0	0	0.000	0.000	-0.006
GSM1424161_Control	0.075	0	0	0.185	0.013	0	0.213	0	0	0.055	0.208	0.015	0	0.104	0.015	0	0.005	0	0.112	0	0	0.000	0.000	0.399
GSM1424162_Control	0	0.019	0	0.17	0.108	0	0.093	0.003	0.079	0	0.071	0.115	0	0.08	0.029	0	0.002	0	0.23	0	0	0.000	0.000	0.039
GSM1424163_Control	0	0.003	0.183	0	0.215	0	0.151	0	0.11	0	0	0.052	0	0.069	0	0	0	0.003	0.214	0	0	0.000	0.000	0.042
GSM1424164_Control	0.076	0	0	0	0	0.09	0.026	0.127	0	0	0.019	0.196	0	0	0	0	0	0	0.322	0	0.145	0.000	0.000	-0.006
GSM1424165_Control	0	0.208	0.136	0	0.186	0	0.05	0	0.016	0.026	0	0.033	0	0.015	0	0	0.023	0	0.187	0	0.123	0.000	0.000	0.046
GSM1424166_Control	0.003	0.041	0.036	0.125	0	0	0.186	0	0	0.164	0	0.086	0	0.109	0	0	0.08	0	0.169	0	0	0.000	0.000	0.151
GSM1424167_Control	0	0.141	0.04	0.068	0	0.015	0.086	0	0	0.049	0.055	0	0	0	0.022	0.267	0.018	0.046	0.065	0	0	0.129	0.000	0.213
GSM1424168_Control	0.046	0	0.04	0.331	0	0	0.075	0.042	0.05	0	0	0.106	0	0.14	0	0	0	0	0.089	0	0.081	0.000	0.000	-0.051
GSM1424169_Control	0	0.172	0.036	0	0	0.271	0.046	0	0	0	0	0.011	0	0	0	0.158	0.12	0.003	0.118	0	0.065	0.000	0.000	0.069
GSM1424170_Control	0.021	0.012	0	0	0	0.167	0	0	0.006	0.061	0.028	0.025	0.183	0	0.032	0.048	0.031	0	0.103	0	0.008	0.276	0.000	0.446
GSM1424171_Control	0	0.038	0.143	0.054	0.134	0.026	0.026	0	0.072	0	0.072	0.102	0	0.018	0	0	0	0	0.212	0	0.104	0.000	0.000	0.044
GSM1424172_Control	0.187	0	0	0	0.15	0.006	0.122	0	0.043	0	0.287	0	0	0	0.003	0	0	0	0.17	0	0.031	0.001	0.000	0.147
GSM1424173_Control	0.078	0	0	0	0.207	0	0	0	0.022	0	0.067	0	0.111	0.03	0.026	0.129	0.048	0.02	0.143	0	0	0.120	0.000	0.165
GSM1424174_Control	0	0	0.157	0.096	0	0.04	0.003	0.102	0.076	0.132	0	0.12	0	0	0	0	0.004	0	0.224	0	0.046	0.000	0.000	0.048
GSM1424175_Control	0	0	0.066	0	0	0.15	0	0.124	0.069	0.04	0	0.123	0	0	0.034	0	0	0.017	0	0.294	0.083	0.000	0.000	0.168
GSM1424176_Control	0	0.024	0.194	0.356	0	0	0.048	0.084	0	0	0	0.08	0	0.123	0	0	0	0	0.064	0	0.027	0.000	0.000	-0.003
GSM1424177_Control	0	0	0.167	0	0.112	0.1	0.141	0	0.133	0.1	0	0.074	0	0	0.071	0	0.031	0	0.07	0	0	0.000	0.000	0.057
GSM1424178_Control	0	0	0.11	0	0	0.385	0	0	0	0	0	0.163	0	0.127	0.022	0	0	0.031	0.149	0	0.034	0.000	0.000	0.069
GSM1424179_Control	0	0.182	0.11	0.225	0.002	0	0.099	0	0.035	0.175	0	0.073	0	0	0.012	0	0.085	0	0.002	0	0	0.000	0.000	0.062
GSM1424180_Control	0	0.011	0.21	0.017	0.032	0.177	0.104	0	0	0	0	0.207	0	0	0.032	0	0.176	0	0	0.034	0	0.000	0.000	0.064
GSM1424181_Control	0	0	0.01	0.189	0	0	0.127	0.039	0	0.14	0	0.059	0	0.01	0.075	0	0.098	0	0.12	0	0.134	0.000	0.000	-0.001
GSM1424182_Control	0	0	0.09	0.24	0	0	0.172	0.03	0	0	0.098	0.034	0	0	0	0	0.072	0	0.221	0	0.043	0.000	0.000	0.033
GSM1424183_Control	0	0.121	0.123	0.253	0	0	0.14	0	0.03	0.076	0	0	0	0.08	0	0	0	0	0.162	0	0.014	0.000	0.000	0.013
GSM1424184_Control	0	0.06	0.18	0.311	0	0	0.058	0	0.185	0	0	0	0	0.106	0	0	0	0.015	0.085	0	0	0.000	0.000	-0.042

Input Sample	B cells naive	B cells memory	Plasma cells	T cells CD8	T cells CD4 naive	T cells CD4 memory resting	T cells CD4 memory activated	T cells follicular helper	T cells regulatory (Tregs)	T cells gamma delta	NK cells resting	NK cells activated	Monocytes	Macrophages M0	Macrophages M1	Macrophages M2	Dendritic cells resting	Dendritic cells activated	Mast cells resting	Mast cells activated	Eosinophils	Neutrophils	P-value	Correlation
GSM1424185_Control	0	0.017	0	0.075	0	0	0.235	0	0	0	0.318	0	0	0	0.12	0	0.1	0	0.11	0	0.025	0.000	0.000	0.033
GSM1424186_Control	0	0.159	0.134	0.132	0.171	0	0.215	0	0.078	0	0.067	0	0	0.024	0	0	0	0	0.011	0	0.009	0.000	0.000	0.017
GSM1424187_Control	0	0.114	0.096	0.128	0	0	0.15	0	0.178	0	0	0.072	0	0.08	0	0	0	0	0.182	0	0	0.000	0.000	-0.009
GSM1424188_Control	0	0.064	0.093	0.347	0	0	0.129	0.215	0	0	0	0.058	0	0.038	0.02	0	0	0	0.036	0	0	0.000	0.000	0.061
GSM1424189_Control	0	0.102	0.173	0.255	0.053	0	0	0	0.047	0	0	0.111	0	0.116	0.012	0	0.025	0	0.104	0	0	0.000	0.000	0.012
GSM1424190_Control	0	0.022	0.053	0.35	0.116	0	0.087	0	0.036	0	0	0.035	0	0.136	0	0	0.026	0	0.137	0	0	0.000	0.000	0.004
GSM1424191_Control	0	0.093	0	0.118	0	0	0.082	0.109	0	0.092	0.04	0	0	0	0.09	0	0.078	0	0	0.008	0.29	0.000	0.000	-0.019
GSM1424192_Control	0	0.103	0.084	0.208	0	0	0.108	0	0.035	0	0	0.089	0	0.087	0	0	0	0.025	0.23	0	0.033	0.000	0.000	-0.072
GSM1424193_Control	0	0.062	0.197	0.23	0.083	0	0.18	0	0.031	0.012	0	0.177	0	0.004	0	0	0	0	0.024	0	0	0.000	0.000	0.053
GSM1424194_Control	0	0.002	0.085	0.19	0.092	0	0.196	0	0	0.225	0	0	0	0.085	0	0	0	0	0.125	0	0	0.000	0.000	0.116
GSM1424195_Control	0	0.134	0.011	0.057	0	0.015	0.078	0	0	0	0	0	0	0	0.064	0.203	0.069	0.004	0.237	0	0.004	0.125	0.000	0.196
GSM1424196_Control	0.186	0	0	0	0.115	0.048	0	0	0.049	0	0	0.069	0	0	0	0.098	0.129	0	0.185	0	0.012	0.109	0.000	0.059
GSM1424197_Control	0	0.028	0.083	0.181	0.155	0	0.205	0	0.005	0.062	0	0.037	0	0.103	0	0	0.001	0	0.141	0	0	0.000	0.000	0.022
GSM1424198_Control	0.101	0	0	0	0.023	0.088	0	0	0	0	0.064	0.015	0.345	0.052	0.015	0.039	0	0	0.106	0	0	0.152	0.000	0.459
GSM1424199_Control	0	0.157	0.054	0.262	0.078	0	0.119	0.038	0	0	0	0.087	0	0.04	0.006	0	0.075	0	0.085	0	0	0.000	0.000	-0.074
GSM1424200_Control	0.061	0	0.02	0.059	0.098	0	0.158	0	0	0	0	0.101	0	0	0.01	0	0.073	0	0.364	0	0.057	0.000	0.000	-0.082
GSM1424201_Control	0	0.144	0.094	0.066	0.027	0	0.178	0.05	0	0.155	0	0.088	0	0.04	0	0	0	0	0.061	0	0.097	0.000	0.000	0.098
GSM1424202_Control	0	0.076	0.014	0.249	0	0.103	0.078	0	0.019	0	0.051	0.143	0	0.067	0	0	0	0	0.2	0	0	0.000	0.000	0.000
GSM1424203_Control	0	0.154	0.026	0.094	0	0.11	0	0.092	0	0	0	0.145	0	0	0.001	0	0.075	0	0.255	0	0.049	0.000	0.000	-0.016
GSM1424204_Control	0.111	0	0	0.329	0	0	0	0	0	0	0	0.197	0	0	0	0	0.083	0	0.222	0	0.059	0.000	0.000	-0.054
GSM1424205_Control	0.043	0.006	0	0.31	0	0	0.083	0.026	0	0.068	0	0.101	0	0	0.02	0.024	0.01	0	0	0.234	0.074	0.000	0.000	0.154
GSM1424206_Control	0	0	0.113	0.255	0.018	0	0.186	0.105	0	0	0	0.069	0	0.039	0	0.025	0	0	0.19	0	0	0.000	0.000	-0.012
GSM1424207_Control	0	0	0.063	0.432	0	0	0.159	0	0	0	0	0.057	0	0.04	0	0	0.101	0	0.113	0	0.035	0.000	0.000	-0.005
GSM1424208_Control	0	0	0.224	0.216	0.125	0	0.186	0	0.068	0	0.01	0	0	0.119	0	0	0	0	0.052	0	0	0.000	0.000	-0.011
GSM1424209_Control	0.08	0	0	0.106	0	0.014	0.166	0	0.049	0	0.125	0	0	0.153	0.008	0	0.106	0	0.19	0	0.003	0.000	0.000	-0.025
GSM1424210_Control	0	0.11	0	0.021	0	0.237	0.058	0.023	0.019	0	0.143	0	0.07	0	0.002	0.081	0.053	0	0.053	0.042	0.089	0.000	0.000	0.144
GSM1424211_Control	0.142	0	0	0.073	0.095	0	0.07	0	0	0.076	0	0.081	0	0.168	0.039	0	0.046	0	0.134	0	0.005	0.071	0.000	0.067
GSM1424212_Control	0	0.251	0.052	0.21	0	0	0.054	0.108	0	0	0	0.19	0	0	0.004	0	0.056	0	0.072	0	0.003	0.000	0.000	0.118
GSM1424213_Control	0.158	0.017	0	0.069	0.134	0.126	0	0	0	0	0.21	0	0	0.176	0	0	0.093	0	0	0	0	0.017	0.000	0.078
GSM1424214_Control	0.128	0	0	0.113	0.046	0	0.122	0.056	0	0.023	0	0.151	0	0.092	0.002	0	0.034	0	0.232	0	0.002	0.000	0.000	0.134
GSM1424215_Control	0	0.12	0	0	0.22	0.05	0	0	0	0.107	0.023	0	0.07	0.03	0	0.027	0.035	0	0	0.102	0	0.215	0.000	0.201
GSM1424216_Control	0.055	0	0	0	0.154	0.057	0	0	0	0.076	0.112	0	0.108	0.149	0.016	0	0	0	0.039	0	0	0.235	0.000	0.296
GSM1424217_Control	0.042	0.043	0	0.123	0	0.078	0.076	0.031	0	0.154	0.015	0.032	0	0.177	0.001	0.021	0.05	0	0.038	0	0.118	0.000	0.000	0.165
GSM1424218_Control	0.226	0	0	0.07	0.02	0.034	0.09	0	0.077	0	0	0.117	0	0.174	0.043	0	0.011	0	0.138	0	0	0.000	0.000	0.068
GSM1424219_Control	0	0.2	0	0	0.218	0.075	0.036	0.007	0	0.108	0.001	0	0	0.207	0.149	0	0	0	0	0	0	0.000	0.000	0.020
GSM1424220_Control	0.172	0	0	0	0.187	0	0.025	0.081	0	0	0.058	0.029	0	0.211	0.021	0.034	0	0.049	0.133	0	0	0.000	0.000	-0.003
GSM1424221_Control	0	0.162	0.002	0.209	0	0	0.073	0.049	0.035	0	0	0.117	0	0.121	0.011	0	0	0	0.175	0	0.046	0.000	0.000	0.085
GSM1424222_Control	0	0.148	0	0.07	0.255	0	0.063	0	0.001	0	0.014	0.014	0	0.037	0	0.026	0.073	0	0	0.066	0	0.233	0.000	0.127
GSM1424223_Control	0	0.185	0	0.16	0.065	0	0.074	0.14	0.019	0	0	0.042	0.013	0.013	0	0.169	0.024	0	0.068	0	0.026	0.000	0.000	0.013
GSM1424224_Control	0	0	0.183	0.175	0.085	0	0.054	0.095	0.021	0	0	0.063	0	0.162	0	0	0.002	0	0.162	0	0	0.000	0.000	0.017
GSM1424225_Control	0	0.089	0.058	0.197	0	0.054	0.118	0	0	0	0	0.164	0	0.002	0.002	0	0	0	0.302	0	0.017	0.000	0.000	0.067
GSM1424226_Control	0	0.083	0.062	0.169	0.034	0	0.064	0.057	0.086	0	0	0.136	0	0.099	0.027	0	0	0	0.183	0	0	0.000	0.000	-0.008
GSM1424227_Control	0	0.186	0	0.218	0.059	0	0.007	0	0.108	0	0	0.062	0	0.068	0	0.129	0	0.014	0.15	0	0	0.000	0.000	0.067
GSM1424228_Control	0	0.161	0.113	0.043	0	0	0.232	0.026	0.104	0.124	0	0	0	0.084	0.042	0	0	0	0.07	0	0	0.000	0.000	-0.002
GSM1424229_Control	0	0.109	0.011	0.237	0	0.115	0.073	0	0	0	0	0.139	0	0.052	0	0	0	0.032	0.153	0	0.079	0.000	0.000	-0.049
GSM1424230_Control	0	0.016	0.015	0.455	0	0	0.223	0	0	0	0	0.003	0	0.007	0.051	0.039	0.153	0	0	0.012	0.026	0.000	0.000	0.145
GSM1424231_Control	0	0.047	0	0.185	0	0	0	0.153	0	0	0	0.111	0	0	0	0	0	0	0	0.465	0.039	0.000	0.000	0.317
GSM1424232_Control	0.023	0.019	0	0.197	0	0.029	0.159	0.014	0	0.069	0	0.127	0	0.087	0	0	0.015	0	0.206	0	0.054	0.000	0.000	0.145
GSM1424233_Control	0	0.099	0	0	0.299	0	0.175	0.006	0	0.159	0	0.017	0	0.144	0	0	0	0	0.101	0	0	0.000	0.000	0.080
GSM1424234_Control	0	0.154	0	0.21	0.012	0	0.164	0	0	0.029	0	0.123	0	0.123	0.001	0.016	0.083	0	0.056	0	0.029	0.000	0.000	0.175
GSM1424235_Control	0	0.209	0.041	0.25	0	0	0.108	0	0.057	0	0	0	0	0.045	0	0.108	0.055	0	0.122	0	0.004	0.000	0.000	0.103
GSM1424236_Control	0	0.061	0.1	0.264	0	0	0.096	0.066	0.08	0.05	0	0.046	0	0.129	0	0	0.075	0	0.033	0	0	0.000	0.000	0.110
GSM1424237_Control	0	0.214	0	0.294	0	0	0.073	0	0.034	0.019	0	0.039	0	0.141	0	0	0	0	0.167	0	0.019	0.000	0.000	0.126
GSM1424238_Control	0	0.322	0	0.192	0.016	0	0.084	0.034	0.008	0	0.044	0.056	0	0.078	0	0.085	0.016	0	0.052	0	0.013	0.000	0.000	0.086
GSM1424239_Control	0	0.028	0.061	0.263	0.13	0	0.274	0.129	0	0.064	0	0.031	0	0	0	0.017	0.003	0	0	0	0	0.000	0.000	0.049
GSM1424240_Control	0	0.289	0.018	0.232	0	0	0.054	0.069	0.008	0	0	0.084	0	0	0	0	0.062	0	0.184	0	0	0.000	0.000	0.056
GSM1424241_Control	0	0	0.1	0.117	0	0.025	0.413	0.017	0	0.018	0	0	0	0.16	0	0	0.008	0	0.142	0	0	0.000	0.000	0.010
GSM1424242_Control	0	0.193	0.1	0.002	0	0.123	0.146	0.124	0	0.1	0	0	0	0.069	0.039	0	0	0	0.1	0	0.003	0.000	0.000	0.105
GSM1424243_Control	0	0.298	0.031	0.236	0.099	0	0.097	0.032	0	0	0	0.009	0	0.133	0	0	0.015	0	0	0.036	0.014	0.000	0.000	0.061
GSM1424244_Control	0	0.178	0.029	0.369	0.013	0	0.081	0	0.068	0	0.014	0	0	0.089	0	0	0	0	0.079	0.02	0	0.060	0.000	-0.041
GSM1424245_Control	0	0.016	0.09	0.162	0	0.151	0.084	0	0	0.138	0	0.068	0	0	0.064	0.047	0.078	0	0	0.086	0.017	0.000	0.000	0.185
GSM1424246_Control	0	0.023	0.132	0.212	0.061	0	0.132	0.007	0	0	0	0.088	0	0.06	0.013	0.046	0.05	0	0.175	0	0	0.000	0.000	-0.017
GSM1423780_Treat	0.029	0	0.031	0	0.121	0	0.058	0	0.07	0	0.029	0.068	0.091	0.061	0.091	0.111	0.02	0	0.201	0	0.018	0.000	0.000	0.089
GSM1423781_Treat	0.037	0	0.02	0.063	0.02	0	0	0	0.235	0	0.095	0	0.261	0	0	0	0.028	0	0.169	0.033	0.038	0.000	0.000	0.028
GSM1423782_Treat	0	0.138	0.085	0.219	0	0.069	0.116	0.048	0	0	0	0.124	0	0.004	0.032	0	0.075	0	0.03	0.059	0	0.000	0.000	0.125
GSM1423783_Treat	0.208	0	0	0	0.115	0.074	0	0.003	0	0	0.085	0.003	0.038	0	0.013	0.21	0	0	0.175	0	0	0.075	0.000	0.039
GSM1423784_Treat	0	0.096	0	0.027	0	0.003	0.068	0.17	0	0	0	0.174	0	0	0	0.05	0.115	0	0.194	0	0.103	0.000	0.000	-0.044
GSM1423785_Treat	0.102	0	0	0.046	0	0.283	0	0	0	0.094	0	0.014	0	0.068	0.004	0.096	0	0	0.294	0	0	0.000	0.000	0.176
GSM1423786_Treat	0.042	0	0	0	0	0.069	0.098	0.012	0.007	0.336	0	0	0.002	0	0	0.208	0.01	0.029	0.125	0	0	0.063	0.000	0.331
GSM1423787_Treat	0.013	0.024	0	0.005	0	0.188	0.065	0	0	0.111	0	0	0	0.06	0.018	0.36	0.001	0.018	0.109	0	0	0.028	0.000	0.336
GSM1423788_Treat	0.112	0	0	0	0.106	0.006	0	0.07	0	0.133	0	0	0	0.189	0.037	0	0	0	0.236	0	0	0.110	0.000	0.055
GSM1423789_Treat	0.05	0	0	0	0.093	0.041	0.04	0	0	0.082	0.084	0	0.329	0.02	0	0.089	0.023	0.008	0.071	0	0	0.070	0.000	0.322
GSM1423790_Treat	0	0.093	0.05	0	0	0.268	0.11	0	0	0.109	0	0.071	0	0.109	0.038	0	0	0.037	0.087	0	0.028	0.000	0.000	0.225
GSM1423791_Treat	0.295	0	0	0	0.196	0.121	0	0	0	0.041	0.025	0	0	0.228	0	0	0	0.019	0.075	0	0	0.000	0.000	-0.002
GSM1423792_Treat	0.071	0	0	0	0	0.271	0.017	0.013	0	0	0.098	0	0	0.072	0	0	0	0.104	0.304	0	0	0.049	0.000	-0.060
GSM1423793_Treat	0.078	0	0	0.135	0	0.123	0.086	0	0	0.042	0	0.05	0.028	0.041	0	0.092	0.091	0	0.183	0	0.053	0.000	0.000	0.015
GSM1423794_Treat	0	0.065	0.024	0	0.023	0.311	0.037	0	0	0.146	0.045	0	0	0.03	0.024	0.136	0.063	0.07	0.021	0	0.005	0.000	0.000	0.159
GSM1423795_Treat	0.006	0	0	0	0.012	0.116	0.031	0	0	0.117	0	0.017	0.023	0	0.056	0.288	0.051	0	0.124	0	0	0.158	0.000	0.297
GSM1423796_Treat	0	0.169	0.004	0	0.122	0.131	0	0.008	0	0.051	0.039	0	0.042	0.056	0.032	0.091	0.128	0.025	0.1	0	0	0.000	0.000	0.145
GSM1423797_Treat	0	0.168	0.047	0.032	0	0.027	0.041	0.078	0	0.231	0	0.003	0	0	0.072	0.201	0.008	0	0.068	0	0.025	0.000	0.000	0.453
GSM1423798_Treat	0.078	0	0	0	0.068	0.076	0.129	0.018	0	0.046	0.02	0	0.028	0.117	0	0.055	0.05	0.058	0.258	0	0	0.000	0.000	0.035
GSM1423799_Treat	0.104	0.025	0	0	0.136	0.062	0.022	0	0	0	0.194	0	0.129	0.069	0.034	0.031	0	0	0.142	0	0.013	0.040	0.000	0.129
GSM1423800_Treat	0.232	0	0	0	0	0.291	0	0	0.001	0.01	0.179	0	0.178	0	0.076	0	0.028	0	0.006	0	0	0.000	0.000	0.099
GSM1423801_Treat	0.062	0.101	0	0	0.027	0.043	0.053	0	0	0.013	0.132	0	0.072	0.059	0.015	0.12	0.014	0	0.144	0	0.039	0.107	0.000	0.272
GSM1423802_Treat	0.033	0.014	0	0	0	0.001	0.022	0	0.005	0.041	0.084	0	0.18	0.141	0	0	0	0.072	0.141	0	0	0.265	0.000	0.383
GSM1423803_Treat	0	0.143	0.029	0	0.048	0.08	0.157	0	0.003	0.107	0.007	0	0	0.083	0	0	0.085	0.156	0.103	0	0	0.000	0.000	0.059
GSM1423804_Treat	0	0.011	0.011	0	0	0.042	0	0.05	0	0.037	0	0.064	0.224	0.015	0.095	0.172	0.027	0.001	0.137	0	0	0.113	0.000	0.350
GSM1423805_Treat	0	0.068	0	0.01	0	0.081	0.031	0.003	0	0	0.089	0	0.171	0	0	0.19	0.024	0.018	0.054	0	0.012	0.248	0.000	0.334
GSM1423806_Treat	0.136	0	0	0	0.025	0.205	0.024	0	0	0.002	0.08	0	0.127	0.111	0.044	0.096	0	0	0.073	0	0.017	0.059	0.000	0.144
GSM1423807_Treat	0.082	0	0	0	0.023	0.084	0.138	0	0	0.162	0	0	0.021	0.051	0	0.157	0	0.048	0.16	0	0	0.074	0.000	0.151
GSM1423808_Treat	0.028	0	0	0	0.05	0.019	0.069	0	0	0.161	0	0.025	0.116	0.017	0.022	0.144	0.144	0	0.113	0	0.021	0.071	0.000	0.193
GSM1423809_Treat	0.119	0	0	0	0.328	0	0.058	0.111	0	0.111	0	0	0	0.125	0	0	0.111	0	0	0	0.037	0.000	0.000	-0.062
GSM1423810_Treat	0.099	0	0	0	0.026	0.06	0.099	0.056	0	0.081	0.011	0	0	0.055	0	0.118	0.017	0.082	0.157	0	0	0.139	0.000	0.200
GSM1423811_Treat	0.048	0	0	0	0.133	0.025	0.013	0	0.071	0.127	0	0	0.001	0.022	0	0.156	0.126	0	0.163	0	0.114	0.000	0.000	0.105
GSM1423812_Treat	0	0.056	0.027	0	0.142	0.205	0	0	0	0	0.219	0	0	0.069	0	0.106	0	0.13	0.046	0	0	0.000	0.000	0.071
GSM1423813_Treat	0.287	0	0	0	0.147	0	0.018	0.029	0	0	0.22	0	0	0.025	0.062	0.06	0.061	0	0.09	0	0	0.000	0.000	0.084
GSM1423814_Treat	0	0.021	0.006	0	0.297	0	0.063	0.027	0	0.041	0	0.084	0	0	0	0	0.144	0	0.316	0	0	0.000	0.000	0.006

Input Sample	B cells naive	B cells memory	Plasma cells	T cells CD8	T cells CD4 naive	T cells CD4 memory resting	T cells CD4 memory activated	T cells follicular helper	T cells regulatory (Tregs)	T cells gamma delta	NK cells resting	NK cells activated	Monocytes	Macrophages M0	Macrophages M1	Macrophages M2	Dendritic cells resting	Dendritic cells activated	Mast cells resting	Mast cells activated	Eosinophils	Neutrophils	P-value	Correlation
GSM1423815_Treat	0	0.159	0.034	0	0	0.258	0.008	0	0.013	0	0	0	0	0.066	0	0.159	0	0.009	0.295	0	0	0.000	0.000	0.052
GSM1423816_Treat	0.229	0	0	0	0.093	0	0	0.262	0	0	0.064	0.025	0	0.008	0	0	0.013	0	0.225	0	0.082	0.000	0.000	-0.013
GSM1423817_Treat	0.024	0	0	0.059	0	0.107	0.088	0.006	0	0.146	0	0	0.029	0	0.03	0.259	0.054	0.01	0	0.047	0.004	0.135	0.000	0.370
GSM1423818_Treat	0	0.061	0	0.009	0	0.066	0.028	0.04	0	0.082	0	0	0	0	0	0.215	0.097	0.018	0.168	0	0	0.216	0.000	0.230
GSM1423819_Treat	0.187	0	0	0.027	0	0.09	0.219	0	0	0.069	0.103	0	0.08	0.05	0	0.02	0	0.079	0.075	0	0	0.000	0.000	0.180
GSM1423820_Treat	0	0.036	0	0	0	0.282	0.014	0	0	0.156	0	0.039	0.028	0	0.04	0.171	0.171	0.004	0	0.028	0	0.031	0.000	0.242
GSM1423821_Treat	0.124	0	0	0	0	0.292	0	0	0.036	0.003	0.003	0.031	0.082	0	0.004	0.182	0.022	0.036	0	0.158	0.026	0.000	0.000	0.193
GSM1423822_Treat	0	0.063	0.024	0.097	0	0	0.042	0.138	0	0.047	0	0.279	0	0.066	0	0	0.116	0.026	0	0	0.103	0.000	0.000	0.064
GSM1423823_Treat	0.105	0	0	0	0.13	0.214	0	0	0.022	0.157	0.003	0	0.057	0.081	0.034	0.074	0.023	0	0.1	0	0	0.000	0.000	0.109
GSM1423824_Treat	0.058	0	0	0	0.027	0.231	0	0	0	0.161	0.03	0	0.256	0	0.007	0	0	0	0.122	0	0	0.108	0.000	0.218
GSM1423825_Treat	0	0.19	0.013	0.009	0.028	0.19	0.039	0.016	0	0	0.068	0	0	0	0	0.138	0.075	0.018	0.037	0	0	0.178	0.000	0.201
GSM1423826_Treat	0	0.046	0	0	0.003	0.132	0.03	0	0	0.022	0.044	0	0.248	0	0.074	0.205	0.002	0	0.097	0	0	0.097	0.000	0.312
GSM1423827_Treat	0	0	0.002	0	0.087	0.114	0.057	0	0	0	0.092	0	0.288	0.064	0.057	0.01	0	0.025	0.095	0	0.012	0.099	0.000	0.272
GSM1423828_Treat	0	0.117	0	0	0	0.065	0.027	0	0	0.043	0.06	0	0.244	0	0	0.115	0.031	0.021	0.066	0	0	0.210	0.000	0.414
GSM1423829_Treat	0	0.076	0	0	0	0.064	0.034	0	0.07	0.055	0.053	0	0.18	0.04	0.073	0.02	0.014	0	0.123	0	0	0.197	0.000	0.305
GSM1423830_Treat	0.179	0	0	0.477	0	0	0.152	0.042	0	0	0	0	0	0.064	0	0	0.008	0	0.078	0	0	0.000	0.000	0.092
GSM1423831_Treat	0	0.135	0	0	0.122	0.086	0	0	0.036	0	0.3	0	0	0.16	0.036	0	0	0	0.078	0	0.025	0.022	0.000	0.062
GSM1423832_Treat	0.161	0	0	0	0.209	0.197	0	0	0	0.07	0.024	0	0	0.177	0	0	0.041	0.033	0.087	0	0	0.000	0.000	0.034
GSM1423833_Treat	0.094	0	0	0.043	0	0.198	0.082	0.134	0	0.012	0.082	0.025	0	0.076	0.056	0	0	0.039	0.158	0	0	0.000	0.000	0.180
GSM1423834_Treat	0.083	0.015	0	0.007	0.023	0.182	0.063	0	0	0	0.006	0.102	0	0.051	0	0.286	0.012	0	0.169	0	0	0.000	0.000	0.096
GSM1423835_Treat	0	0.08	0.02	0	0	0.075	0.076	0	0	0.088	0	0	0.072	0	0	0.224	0.046	0.061	0.138	0	0	0.119	0.000	0.271
GSM1423836_Treat	0.115	0.016	0	0	0	0	0.139	0.148	0	0	0.007	0.017	0	0.094	0.013	0.053	0.056	0	0.291	0	0.05	0.000	0.000	0.026
GSM1423837_Treat	0.106	0	0	0	0	0.236	0.103	0	0	0	0.173	0	0.048	0	0	0.097	0.038	0.028	0	0.039	0.019	0.111	0.000	0.133
GSM1423838_Treat	0.15	0	0	0.08	0	0.253	0.268	0	0	0	0	0	0	0	0	0.164	0	0.054	0.031	0	0	0.000	0.000	-0.101
GSM1423839_Treat	0	0.036	0.006	0	0.021	0.15	0	0.076	0	0.068	0	0.085	0	0	0	0.189	0.007	0.033	0	0.328	0	0.000	0.000	0.188
GSM1423840_Treat	0.061	0	0	0.072	0	0	0.015	0.052	0	0	0.023	0.021	0.125	0.088	0.117	0	0.014	0	0.181	0	0.022	0.208	0.000	0.386
GSM1423841_Treat	0.128	0	0	0.017	0.124	0	0.093	0.05	0	0.073	0	0	0	0.023	0	0	0.1	0.055	0.328	0	0.009	0.000	0.000	-0.001
GSM1423842_Treat	0.14	0	0	0	0.281	0.044	0	0.027	0	0.044	0.131	0	0.042	0.016	0.009	0	0	0.02	0.212	0	0	0.036	0.000	0.030
GSM1423843_Treat	0.172	0	0	0	0.202	0	0	0	0	0.052	0	0.021	0.068	0.14	0.024	0	0	0	0.26	0	0	0.060	0.000	0.122
GSM1423844_Treat	0.176	0	0	0	0.002	0.23	0.059	0	0	0.043	0.088	0	0	0	0.025	0.033	0	0	0.147	0	0.008	0.188	0.000	0.127
GSM1423845_Treat	0	0.075	0	0	0.021	0.165	0.042	0	0	0.016	0.122	0	0	0	0	0.234	0.018	0.064	0.169	0	0.008	0.067	0.000	0.156
GSM1423846_Treat	0.159	0	0	0	0	0.155	0	0	0.017	0.085	0	0	0	0	0	0.167	0.046	0	0	0.316	0.023	0.033	0.000	0.290
GSM1423847_Treat	0.004	0.061	0	0	0	0.082	0	0	0.027	0	0.014	0.001	0.042	0	0.023	0.409	0.071	0	0.185	0	0.023	0.058	0.000	0.381
GSM1423848_Treat	0.085	0	0	0	0.123	0	0	0.027	0	0	0.08	0.005	0.196	0.006	0.012	0	0	0	0.152	0	0	0.313	0.000	0.301
GSM1423849_Treat	0	0.125	0	0	0.093	0	0	0	0	0	0	0.039	0.149	0	0	0.265	0.068	0.003	0.106	0	0	0.153	0.000	0.242
GSM1423850_Treat	0.019	0	0	0	0	0.176	0.028	0	0	0.047	0.024	0	0	0.004	0.053	0.173	0.011	0.048	0.268	0	0	0.148	0.000	0.157
GSM1423851_Treat	0.022	0.036	0	0	0	0.2	0.063	0	0	0.223	0	0	0.004	0.019	0	0.111	0.05	0.009	0.142	0	0	0.120	0.000	0.269
GSM1423852_Treat	0.022	0	0.034	0	0.165	0	0	0.024	0.072	0	0	0.12	0.001	0.166	0	0	0	0	0.218	0	0	0.179	0.000	0.109
GSM1423853_Treat	0.111	0	0	0	0.06	0.111	0.005	0	0	0.033	0.186	0	0.052	0	0.014	0.105	0.065	0.043	0.029	0	0.032	0.152	0.000	0.220
GSM1423854_Treat	0.055	0	0	0	0.086	0.307	0.034	0	0	0.123	0.081	0	0	0.07	0.01	0.027	0.024	0.034	0.104	0	0	0.044	0.000	0.151
GSM1423855_Treat	0.091	0	0	0	0	0.297	0	0.002	0	0.149	0	0	0	0	0	0.122	0.144	0	0	0.195	0	0.000	0.000	0.047
GSM1423856_Treat	0	0.091	0	0.014	0	0.142	0.041	0	0.011	0.168	0	0	0.02	0	0	0.216	0.021	0	0.179	0	0.034	0.062	0.000	0.233
GSM1423857_Treat	0.043	0.037	0	0.003	0.009	0.066	0.073	0	0	0.245	0	0	0.036	0.111	0	0.062	0.024	0	0.293	0	0	0.000	0.000	0.124
GSM1423858_Treat	0.067	0.003	0	0	0.091	0.136	0.06	0	0	0	0.209	0	0.076	0.107	0.05	0	0	0.058	0.108	0	0	0.036	0.000	0.105
GSM1423859_Treat	0.101	0	0	0	0.035	0.277	0.025	0	0	0.198	0.051	0	0.031	0.093	0.04	0.024	0.039	0.037	0.049	0	0	0.000	0.000	0.213
GSM1423860_Treat	0.148	0	0	0	0	0.131	0	0.006	0	0	0.129	0	0.036	0.008	0.013	0.242	0.04	0	0.215	0	0.031	0.000	0.000	0.130
GSM1423861_Treat	0	0.157	0	0	0.034	0	0.107	0	0	0.024	0.059	0	0.022	0.034	0.123	0.062	0	0.007	0.15	0	0.024	0.197	0.000	0.282
GSM1423862_Treat	0	0.159	0.04	0.174	0.015	0	0.083	0	0	0	0	0.003	0	0.044	0	0.065	0.17	0.004	0.197	0	0.047	0.000	0.000	0.002
GSM1423863_Treat	0	0.037	0.022	0	0.044	0	0.076	0	0	0.148	0	0	0.16	0.046	0.082	0.035	0.004	0	0.186	0	0.011	0.150	0.000	0.351
GSM1423864_Treat	0	0.018	0	0.015	0	0.126	0.022	0	0.047	0.022	0	0.045	0	0	0.029	0.313	0.062	0	0	0.118	0	0.185	0.000	0.250
GSM1423865_Treat	0.158	0	0	0	0	0.257	0.101	0	0	0	0.005	0.015	0	0	0.024	0.132	0.076	0	0.165	0	0.049	0.018	0.000	0.028
GSM1423866_Treat	0.112	0	0	0	0.164	0.023	0	0	0	0	0.091	0	0.042	0	0.02	0.223	0	0	0.117	0	0	0.208	0.000	0.282
GSM1423867_Treat	0	0.08	0.034	0.119	0	0	0.005	0	0.151	0	0.019	0.047	0	0.051	0	0.098	0.145	0	0.218	0	0.033	0.000	0.000	0.010
GSM1423868_Treat	0.13	0	0	0	0.033	0.132	0.073	0.026	0	0.146	0.018	0	0.037	0.126	0	0	0	0.024	0.219	0	0.012	0.023	0.000	0.073
GSM1423869_Treat	0.056	0	0	0.033	0	0.12	0.125	0	0.038	0	0.146	0	0.037	0.002	0	0.119	0.019	0.1	0	0.066	0.06	0.078	0.000	0.147
GSM1423870_Treat	0.025	0	0	0	0.201	0.101	0.065	0	0	0.007	0.154	0	0	0	0.022	0	0.1	0.007	0.225	0	0.011	0.081	0.000	0.021
GSM1423871_Treat	0	0.075	0.005	0	0.214	0.189	0	0	0	0	0.155	0.042	0.071	0.123	0.043	0	0	0	0.002	0	0.032	0.048	0.000	0.096
GSM1423872_Treat	0.149	0	0	0	0.071	0.163	0	0.027	0	0.143	0.1	0	0.069	0.012	0.062	0	0.004	0.033	0.079	0	0	0.088	0.000	0.179
GSM1423873_Treat	0.151	0	0	0	0	0	0.034	0	0.039	0.311	0	0	0.068	0	0	0.176	0.088	0	0.062	0	0	0.070	0.000	0.271
GSM1423874_Treat	0.081	0	0	0	0.125	0.136	0	0	0.053	0	0	0.114	0.103	0.143	0	0	0.037	0.042	0.166	0	0	0.000	0.000	0.045
GSM1423875_Treat	0	0.117	0.01	0	0	0	0	0.026	0.068	0.1	0	0.102	0.032	0	0	0.038	0	0.034	0	0.383	0.018	0.072	0.000	0.247
GSM1423876_Treat	0	0.22	0.074	0.091	0.019	0	0.004	0	0.085	0	0	0.06	0	0.075	0	0.068	0.118	0	0.158	0	0.028	0.000	0.000	0.107
GSM1423877_Treat	0.024	0	0	0	0	0.029	0.16	0.092	0	0.089	0	0.053	0.071	0	0.178	0.098	0.018	0	0.077	0	0	0.111	0.000	0.470
GSM1423878_Treat	0.01	0.09	0	0	0.063	0.086	0.072	0	0	0	0.14	0	0.125	0.05	0.048	0	0.104	0	0.112	0	0.028	0.075	0.000	0.223
GSM1423879_Treat	0	0.032	0	0	0.071	0.095	0.007	0	0	0.054	0.093	0.036	0.108	0.143	0.037	0	0	0	0.128	0	0.005	0.191	0.000	0.243
GSM1423880_Treat	0.094	0.106	0	0	0.351	0	0	0.125	0	0	0.005	0.038	0	0	0	0.046	0.056	0	0.124	0	0.056	0.000	0.000	0.027
GSM1423881_Treat	0.203	0	0	0.002	0.136	0	0.07	0	0.008	0	0.126	0	0.014	0	0.015	0.15	0.07	0	0.136	0	0.07	0.000	0.000	0.098
GSM1423882_Treat	0	0.093	0	0	0	0.135	0.111	0.039	0	0.221	0	0	0	0.031	0	0.084	0.032	0.025	0.189	0	0	0.039	0.000	0.194
GSM1423883_Treat	0.122	0	0	0	0.04	0.059	0.182	0	0.018	0.089	0.158	0	0.074	0.032	0	0.029	0.119	0.005	0	0.017	0	0.055	0.000	0.043
GSM1423884_Treat	0.009	0	0.108	0.132	0	0	0.239	0	0.091	0.189	0	0	0	0.122	0	0	0	0	0	0.11	0	0.000	0.000	0.065
GSM1423885_Treat	0.206	0	0	0	0.301	0.126	0.008	0.005	0	0	0.125	0	0	0	0	0.019	0	0.032	0.152	0	0	0.025	0.000	-0.039
GSM1423886_Treat	0	0.055	0	0	0.031	0.109	0.129	0	0	0	0.066	0	0	0	0.043	0.344	0.026	0.001	0.063	0	0	0.133	0.000	0.229
GSM1423887_Treat	0.068	0	0	0	0	0.146	0	0	0.118	0	0.117	0	0.084	0	0.097	0	0	0.008	0	0.224	0.001	0.138	0.000	0.186
GSM1423888_Treat	0.287	0	0	0	0	0.21	0	0	0.105	0	0.208	0	0	0.139	0	0	0	0.009	0.028	0	0	0.015	0.000	-0.098
GSM1423889_Treat	0.111	0	0	0.122	0	0.2	0.058	0	0	0	0	0.063	0.005	0.144	0.016	0	0.173	0.002	0.107	0	0	0.000	0.000	0.007
GSM1423890_Treat	0.226	0	0.022	0	0.373	0.032	0	0	0	0	0.162	0	0	0.107	0	0	0.074	0	0	0.004	0	0.000	0.000	0.068
GSM1423891_Treat	0.048	0.005	0	0	0	0.127	0.065	0	0	0.051	0.109	0	0	0	0.037	0.356	0.045	0.004	0.077	0	0.028	0.045	0.000	0.189
GSM1423892_Treat	0	0.173	0.038	0	0.197	0	0.037	0.004	0	0.016	0.134	0.024	0	0.008	0.046	0.224	0.025	0.001	0.07	0	0.004	0.000	0.000	0.159
GSM1423893_Treat	0.197	0	0	0.171	0	0	0.009	0.1	0	0	0	0.015	0	0	0	0.139	0.109	0	0.235	0	0.025	0.000	0.000	0.022
GSM1423894_Treat	0.02	0	0	0	0.02	0.223	0	0	0	0.07	0.05	0	0.22	0	0.009	0.045	0	0	0.035	0.027	0.002	0.280	0.000	0.431
GSM1423895_Treat	0.394	0	0	0	0.119	0.013	0.024	0.061	0	0.041	0.063	0	0	0.097	0.015	0.032	0	0	0.14	0	0	0.000	0.000	0.073
GSM1423896_Treat	0.121	0	0	0	0.079	0.141	0.019	0	0.048	0	0.163	0	0	0	0	0.238	0.023	0.086	0.08	0	0	0.000	0.000	0.093
GSM1423897_Treat	0.146	0	0	0	0	0.477	0	0.003	0	0	0.126	0	0	0.147	0.004	0	0.036	0.013	0.048	0	0	0.000	0.000	0.121
GSM1423898_Treat	0.175	0	0	0	0.06	0.036	0.018	0.101	0	0.092	0	0	0.088	0.025	0	0.18	0.072	0	0.115	0	0.005	0.034	0.000	0.064
GSM1423899_Treat	0	0.267	0	0	0.072	0.137	0	0	0	0	0.103	0	0.103	0	0	0.133	0	0.051	0.129	0	0.007	0.000	0.000	0.083
GSM1423900_Treat	0.183	0	0	0	0.095	0.201	0.001	0.006	0	0.063	0	0.045	0	0.092	0	0	0	0.028	0.287	0	0	0.000	0.000	-0.017
GSM1423901_Treat	0.225	0	0	0	0.117	0	0	0.038	0	0	0.213	0	0.045	0.028	0.052	0.108	0	0	0.092	0	0.081	0.000	0.000	0.054
GSM1423902_Treat	0	0.067	0	0.141	0.138	0.024	0.128	0.106	0	0.046	0.014	0	0	0.009	0	0	0.114	0	0.215	0	0	0.000	0.000	0.052
GSM1423903_Treat	0.185	0	0	0	0.095	0.283	0.05	0	0	0.018	0.066	0	0	0.132	0.01	0.026	0	0.012	0.124	0	0	0.000	0.000	0.013
GSM1423904_Treat	0	0.065	0	0	0	0.183	0.013	0	0	0.01	0.059	0	0.149	0	0	0.104	0.073	0	0.102	0	0	0.242	0.000	0.353
GSM1423905_Treat	0	0.157	0.134	0.232	0	0.062	0.171	0	0	0	0.12	0	0	0.083	0	0	0	0	0.031	0	0.009	0.000	0.000	0.005
GSM1423906_Treat	0.054	0	0.025	0	0.076	0	0.081	0	0	0.201	0.018	0	0.118	0.054	0	0.062	0	0.067	0.068	0	0	0.176	0.000	0.300
GSM1423907_Treat	0	0.033	0	0	0	0.197	0.086	0	0	0.09	0.021	0	0	0.056	0.037	0.276	0.037	0	0.039	0.021	0	0.107	0.000	0.287
GSM1423908_Treat	0	0.054	0	0	0	0.095	0.021	0.049	0	0.006	0.041	0.029	0.227	0.03	0.034	0.017	0.041	0.064	0.093	0	0.023	0.176	0.000	0.312
GSM1423909_Treat	0	0.096	0.108	0	0.239	0.004	0	0.008	0	0	0	0.313	0	0.035	0	0.054	0.083	0	0.042	0	0.018	0.000	0.000	0.063
GSM1423910_Treat	0.117	0	0	0.008	0	0.101	0.142	0	0	0.134	0	0	0	0	0.07	0.095	0.121	0	0.094	0	0	0.116	0.000	0.195
GSM1423911_Treat	0.047	0	0.003	0	0.046	0.245	0.041	0	0	0	0.15	0	0.154	0.066	0.003	0.071	0.003	0	0.13	0	0	0.040	0.000	0.194

Input Sample	B cells naive	B cells memory	Plasma cells	T cells CD8	T cells CD4 naive	T cells CD4 memory resting	T cells CD4 memory activated	T cells follicular helper	T cells regulatory (Tregs)	T cells gamma delta	NK cells resting	NK cells activated	Monocytes	Macrophages M0	Macrophages M1	Macrophages M2	Dendritic cells resting	Dendritic cells activated	Mast cells resting	Mast cells activated	Eosinophils	Neutrophils	P-value	Correlation
GSM1423912_Treat	0.091	0	0	0	0.204	0.054	0.039	0	0	0.008	0.202	0	0	0.149	0.117	0	0.028	0	0.108	0	0	0.000	0.000	0.151
GSM1423913_Treat	0	0.064	0	0	0.083	0.065	0	0	0.006	0.101	0	0.021	0.156	0.028	0.022	0.122	0	0	0.188	0	0	0.146	0.000	0.301
GSM1423914_Treat	0.064	0.078	0	0	0.034	0.074	0.021	0.051	0.019	0	0.284	0	0.001	0.12	0.047	0	0	0	0.207	0	0	0.000	0.000	0.172
GSM1423915_Treat	0.042	0	0	0.055	0.066	0.007	0	0.021	0	0.045	0	0	0.148	0	0.004	0.179	0.107	0	0	0.167	0	0.159	0.000	0.311
GSM1423916_Treat	0.103	0	0.032	0	0.103	0.069	0.054	0.029	0	0.183	0	0	0	0.104	0	0	0.043	0.123	0.155	0	0	0.000	0.000	0.007
GSM1423917_Treat	0.201	0	0	0.083	0.048	0.219	0.01	0	0	0	0	0.054	0	0.091	0	0.045	0	0	0.22	0	0.029	0.000	0.000	0.031
GSM1423918_Treat	0.062	0.003	0	0	0	0.197	0.023	0.079	0	0.014	0.198	0	0.069	0.14	0.021	0	0	0.044	0	0.027	0	0.124	0.000	0.142
GSM1423919_Treat	0.178	0	0	0	0.276	0	0.014	0	0	0	0	0.065	0.045	0	0	0	0	0	0.251	0	0	0.170	0.000	0.137
GSM1423920_Treat	0.143	0	0	0	0.169	0	0.015	0	0	0.004	0.077	0	0.229	0.008	0.057	0	0	0	0.115	0	0.022	0.160	0.000	0.305
GSM1423921_Treat	0.172	0	0	0	0	0.286	0.135	0	0	0.125	0.124	0	0	0.101	0	0	0	0.046	0	0.012	0	0.000	0.000	0.142
GSM1423922_Treat	0.044	0	0.019	0	0.092	0	0.061	0.009	0	0.071	0.168	0	0.158	0.061	0	0	0	0.006	0.147	0	0	0.164	0.000	0.273
GSM1423923_Treat	0.061	0	0	0	0	0.209	0.004	0	0	0.101	0.023	0	0.207	0.02	0.019	0.084	0.012	0	0.185	0	0	0.075	0.000	0.264
GSM1423924_Treat	0.159	0	0	0	0.209	0	0.062	0	0	0	0.089	0	0.211	0.039	0.003	0	0.017	0	0.132	0	0.014	0.065	0.000	0.261
GSM1423925_Treat	0	0.037	0.115	0	0.171	0	0.167	0	0	0.007	0	0.098	0	0.092	0	0	0.123	0	0	0.132	0.057	0.000	0.000	0.086
GSM1423926_Treat	0.077	0	0	0	0.072	0.306	0.04	0	0	0	0.03	0	0	0	0.006	0.245	0.117	0.021	0.086	0	0	0.000	0.000	0.028
GSM1423927_Treat	0.018	0.099	0	0.043	0.108	0	0.262	0	0	0	0.039	0	0	0.079	0	0.14	0.06	0.024	0.128	0	0	0.000	0.000	0.055
GSM1423928_Treat	0.105	0	0	0	0.012	0.118	0.101	0	0	0.172	0.074	0	0.036	0.046	0.02	0	0	0.049	0.174	0	0	0.094	0.000	0.218
GSM1423929_Treat	0.155	0	0	0.019	0	0.072	0	0.047	0	0	0.036	0	0	0.023	0	0.229	0.052	0.05	0.201	0	0.015	0.101	0.000	0.183
GSM1423930_Treat	0	0.017	0.003	0	0.042	0.139	0.114	0.008	0	0.092	0.211	0	0.007	0.189	0	0	0.031	0.02	0.114	0	0.013	0.000	0.000	0.131
GSM1423931_Treat	0.302	0	0	0	0.168	0.001	0	0	0	0.144	0	0	0	0.144	0	0	0	0.054	0.188	0	0	0.000	0.000	0.019
GSM1423932_Treat	0.24	0	0.017	0	0	0.241	0	0	0.156	0	0.129	0	0	0.081	0.024	0	0.008	0	0.103	0	0	0.000	0.000	-0.003
GSM1423933_Treat	0.074	0	0	0	0.119	0.12	0.005	0	0	0.046	0.093	0	0	0.007	0	0.209	0.041	0	0.172	0	0	0.115	0.000	0.102
GSM1423934_Treat	0	0.063	0	0	0	0.152	0.072	0	0.024	0.15	0.068	0	0	0	0.06	0.11	0.064	0	0.007	0.003	0	0.226	0.000	0.248
GSM1423935_Treat	0.018	0.03	0	0	0.331	0	0	0.122	0	0	0.141	0	0.041	0.104	0	0	0	0	0.079	0	0	0.136	0.000	0.100
GSM1423936_Treat	0	0.221	0.024	0	0.009	0.129	0.154	0	0	0.096	0	0	0	0.049	0	0.091	0.096	0	0.124	0	0.007	0.000	0.000	0.034
GSM1423937_Treat	0.167	0	0	0	0.19	0	0	0	0	0.014	0.142	0	0.122	0.064	0	0	0	0	0.127	0	0	0.174	0.000	0.230
GSM1423938_Treat	0.053	0.005	0	0.054	0	0.083	0	0.037	0	0	0.015	0	0	0	0	0.077	0.004	0	0	0.479	0.059	0.134	0.000	0.294
GSM1423939_Treat	0	0	0.113	0.286	0	0.038	0	0.039	0	0	0	0.098	0	0.167	0	0	0.015	0.036	0.208	0	0	0.000	0.000	-0.000
GSM1423940_Treat	0.23	0	0	0	0	0.249	0	0.038	0	0.033	0	0.058	0.079	0.203	0	0	0	0	0.111	0	0	0.000	0.000	0.026
GSM1423941_Treat	0.378	0	0	0.081	0.163	0	0	0.161	0	0	0.09	0.05	0	0	0	0	0.023	0	0.013	0	0.04	0.000	0.000	-0.016
GSM1423942_Treat	0	0.086	0	0	0	0.047	0.03	0.034	0	0	0	0.067	0.084	0	0.001	0.196	0.115	0	0.191	0	0	0.149	0.000	0.254
GSM1423943_Treat	0	0.104	0	0.085	0.001	0	0.119	0	0	0	0.04	0	0.088	0.023	0.009	0.067	0.103	0.021	0.189	0	0	0.151	0.000	0.087
GSM1423944_Treat	0.039	0	0	0	0.08	0	0.081	0	0	0.029	0.043	0.039	0.04	0	0.037	0.289	0.052	0.018	0.1	0	0.015	0.138	0.000	0.380
GSM1423945_Treat	0.24	0	0	0	0.102	0.156	0.044	0	0	0	0.237	0	0.076	0.044	0.029	0	0	0	0.071	0	0	0.000	0.000	0.055
GSM1423946_Treat	0.198	0	0	0	0	0.199	0.104	0	0	0	0.187	0	0.061	0.069	0	0.068	0	0.01	0.103	0	0	0.000	0.000	0.087
GSM1423947_Treat	0.197	0	0	0.041	0	0.27	0.022	0.145	0	0	0.039	0.054	0	0	0.038	0	0.006	0	0.189	0	0	0.000	0.000	0.033
GSM1423948_Treat	0.27	0	0	0.012	0	0.167	0	0.059	0	0	0.067	0	0	0	0	0.107	0.067	0.084	0.167	0	0	0.000	0.000	-0.035
GSM1423949_Treat	0	0.016	0	0	0.025	0	0.077	0.027	0	0.177	0	0.035	0.008	0.059	0	0.122	0.075	0	0.178	0	0	0.202	0.000	0.242
GSM1423950_Treat	0	0.103	0	0.208	0	0	0.014	0.187	0	0	0	0.063	0	0.195	0	0	0	0	0.157	0	0.072	0.000	0.000	-0.024
GSM1423951_Treat	0.075	0	0	0.02	0.088	0	0.076	0	0.091	0.171	0.02	0	0	0.346	0	0	0	0	0.112	0	0	0.000	0.000	0.163
GSM1423952_Treat	0	0.117	0	0	0.052	0.125	0.109	0	0	0.193	0	0	0.054	0.03	0.007	0.14	0.103	0	0	0	0.013	0.057	0.000	0.234
GSM1423953_Treat	0.064	0	0	0	0.167	0	0.095	0	0	0.322	0	0	0	0.053	0	0.089	0.032	0.016	0.079	0	0	0.083	0.000	0.273
GSM1423954_Treat	0.076	0	0	0.01	0.057	0.089	0.004	0	0	0.108	0	0	0.064	0	0.031	0.317	0.064	0	0.109	0	0.02	0.049	0.000	0.292
GSM1423955_Treat	0	0.16	0.088	0	0.191	0.238	0	0.002	0	0.075	0	0	0	0.014	0	0.111	0	0	0.119	0	0	0.000	0.000	0.043
GSM1423956_Treat	0	0.132	0	0	0.028	0.141	0	0	0.079	0.111	0	0.063	0.056	0.062	0	0.135	0.124	0	0	0.065	0.003	0.000	0.000	0.175
GSM1423957_Treat	0	0.032	0	0.094	0	0.124	0.1	0	0	0.133	0	0	0.068	0.009	0.027	0.107	0.125	0	0.139	0	0.041	0.000	0.000	0.242
GSM1423958_Treat	0.048	0.027	0	0.003	0.083	0	0.094	0	0	0.17	0.004	0	0.091	0.093	0.03	0.051	0.054	0	0.114	0	0	0.137	0.000	0.318
GSM1423959_Treat	0.066	0	0	0	0.243	0.068	0.076	0	0	0.086	0.108	0	0.023	0.168	0	0	0.045	0	0.117	0	0	0.000	0.000	0.130
GSM1423960_Treat	0.118	0	0	0	0.055	0.11	0.078	0	0	0.061	0.047	0.004	0.16	0.078	0	0	0	0	0.108	0	0	0.181	0.000	0.246
GSM1423961_Treat	0.098	0	0	0	0.036	0.191	0.07	0	0	0.124	0.11	0	0.064	0.142	0.018	0	0.027	0.006	0.105	0	0	0.008	0.000	0.237
GSM1423962_Treat	0.146	0.01	0	0	0.131	0.115	0.046	0	0	0	0.069	0.041	0.073	0.152	0.031	0.042	0.016	0	0.087	0	0.041	0.000	0.000	0.155
GSM1423963_Treat	0	0.132	0	0.226	0	0	0.054	0.044	0	0.011	0	0.069	0	0.014	0.004	0.2	0.081	0	0.126	0	0.039	0.000	0.000	0.116
GSM1423964_Treat	0	0.194	0	0	0.131	0.034	0.056	0	0	0.045	0.112	0.025	0.007	0.053	0.026	0.122	0.076	0	0	0.057	0	0.063	0.000	0.213
GSM1423965_Treat	0	0.142	0	0	0.07	0	0.028	0	0.038	0.087	0.145	0	0.124	0.094	0.027	0	0.014	0	0	0.079	0.043	0.110	0.000	0.255
GSM1423966_Treat	0.068	0.005	0	0	0	0.21	0.031	0	0	0.138	0	0.02	0	0.02	0	0.277	0.052	0	0.127	0	0	0.051	0.000	0.087
GSM1423967_Treat	0	0.043	0.005	0.001	0.208	0	0.099	0.001	0	0.195	0	0.065	0	0.137	0	0	0.04	0	0.206	0	0	0.000	0.000	0.042
GSM1423968_Treat	0	0.234	0	0.036	0.023	0.141	0	0	0	0	0.003	0.06	0	0.076	0	0	0.042	0.018	0	0.181	0.021	0.165	0.000	0.093
GSM1423969_Treat	0.038	0.093	0.063	0.16	0.034	0.104	0.022	0	0	0.096	0.076	0	0.021	0.141	0.06	0.006	0.017	0	0.069	0	0	0.000	0.000	0.188
GSM1423970_Treat	0.021	0	0	0	0.104	0	0.009	0	0	0	0.028	0.007	0.286	0.053	0.02	0.255	0.052	0	0.103	0	0	0.062	0.000	0.391
GSM1423971_Treat	0.113	0	0	0	0	0.234	0	0.065	0	0	0.101	0	0.133	0.099	0.145	0	0.045	0	0	0.053	0.012	0.000	0.000	0.139
GSM1423972_Treat	0.025	0.04	0	0	0.099	0.097	0.079	0	0	0.114	0.104	0	0.041	0.015	0	0.193	0.056	0	0.074	0	0	0.063	0.000	0.169
GSM1423973_Treat	0	0.083	0	0.05	0	0.099	0.097	0	0	0.107	0.049	0	0.137	0	0.006	0.131	0.025	0	0	0	0	0.217	0.000	0.440
GSM1423974_Treat	0.153	0	0	0	0	0.299	0.01	0	0	0	0.076	0	0	0.114	0.018	0.1	0.061	0	0.159	0	0.01	0.000	0.000	0.042
GSM1423975_Treat	0.152	0	0	0.144	0	0.076	0.041	0.076	0	0	0.072	0	0	0.203	0.048	0.032	0	0	0.142	0	0.013	0.000	0.000	0.021
GSM1423976_Treat	0	0	0	0	0.2	0	0	0	0.084	0	0.085	0	0.096	0.129	0	0	0.074	0	0.103	0	0	0.229	0.000	0.180
GSM1423977_Treat	0	0.069	0.074	0	0.029	0.2	0	0	0	0	0.101	0.06	0.078	0	0.005	0.14	0.09	0	0.041	0.03	0.082	0.000	0.000	0.162
GSM1423978_Treat	0.039	0.156	0	0.09	0	0.203	0	0	0	0	0.064	0.053	0.005	0.133	0.062	0	0	0	0.157	0	0.04	0.000	0.000	0.093
GSM1423979_Treat	0.074	0	0	0.097	0	0.077	0.105	0	0	0.08	0	0.122	0	0.212	0.062	0	0	0	0.155	0	0.016	0.000	0.000	0.195
GSM1423980_Treat	0	0.107	0.007	0.133	0	0	0	0.157	0	0	0	0.143	0	0.243	0	0	0	0	0.177	0	0.033	0.000	0.000	-0.009
GSM1423981_Treat	0.063	0.074	0	0	0.231	0	0	0	0	0.015	0.145	0.015	0.183	0.192	0.016	0	0	0	0	0	0	0.066	0.000	0.221
GSM1423982_Treat	0.07	0.009	0	0.014	0	0.086	0	0.05	0	0	0.042	0.036	0.27	0.059	0.047	0.209	0	0	0.107	0	0	0.000	0.000	0.280
GSM1423983_Treat	0	0.1	0.018	0	0.026	0.08	0.004	0	0	0.047	0.026	0	0.027	0.042	0.026	0.424	0.014	0	0	0.042	0	0.124	0.000	0.449
GSM1423984_Treat	0.101	0.037	0	0	0.064	0.175	0.001	0	0	0.141	0.075	0	0.009	0.095	0.056	0.11	0	0	0.088	0	0.05	0.000	0.000	0.189
GSM1423985_Treat	0.083	0.049	0	0	0.186	0	0	0	0.007	0.005	0.104	0.029	0.031	0.009	0.031	0.32	0	0	0.132	0	0.013	0.002	0.000	0.234
GSM1423986_Treat	0	0.13	0	0	0.052	0.078	0.073	0	0	0.08	0.033	0.021	0.125	0	0	0.176	0	0	0.022	0	0	0.210	0.000	0.392
GSM1423987_Treat	0.069	0	0	0	0.17	0	0.074	0	0.062	0.063	0	0.08	0	0.205	0	0	0	0	0.235	0	0.043	0.000	0.000	0.034
GSM1423988_Treat	0	0.077	0	0.052	0.083	0	0	0.015	0.028	0.017	0.081	0	0.224	0.062	0.027	0.071	0	0	0	0.235	0.028	0.000	0.000	0.223
GSM1423989_Treat	0.034	0.012	0	0	0.113	0.077	0	0	0	0.148	0.063	0.018	0.142	0.108	0.014	0.159	0	0	0.073	0.027	0	0.011	0.000	0.272
GSM1423990_Treat	0.146	0	0	0	0.372	0	0.002	0	0	0	0.033	0.085	0	0.19	0	0	0	0	0.159	0	0.013	0.000	0.000	0.049
GSM1423991_Treat	0.174	0	0	0.026	0.181	0	0	0	0	0	0.186	0	0.018	0.073	0.039	0.224	0.003	0	0.004	0.001	0.068	0.003	0.000	0.165
GSM1423992_Treat	0.175	0	0	0	0	0.164	0.08	0.019	0	0.078	0.025	0.05	0	0.276	0.026	0	0	0	0.106	0	0	0.000	0.000	0.091
GSM1423993_Treat	0.028	0.028	0	0	0.15	0	0.075	0	0.024	0.061	0.078	0	0.141	0.224	0.008	0	0	0	0.139	0	0	0.043	0.000	0.140
GSM1423994_Treat	0	0.062	0	0.043	0.047	0	0.054	0	0	0.019	0.113	0	0.316	0.052	0	0.13	0	0	0.07	0	0.016	0.080	0.000	0.344
GSM1423995_Treat	0.109	0.032	0	0	0.251	0	0.077	0.019	0	0.05	0.05	0.047	0.025	0.118	0	0	0	0	0.204	0	0.017	0.000	0.000	0.118
GSM1423996_Treat	0	0.019	0	0	0.101	0.026	0.149	0	0	0.21	0.029	0	0.041	0.088	0.099	0.026	0	0	0.108	0	0	0.104	0.000	0.407
GSM1423997_Treat	0.001	0.093	0	0	0.169	0	0	0	0.01	0.001	0.014	0.058	0.057	0.08	0.062	0.351	0	0	0.054	0	0.052	0.000	0.000	0.185
GSM1423998_Treat	0.197	0	0	0.033	0.164	0	0	0	0	0	0.041	0.105	0.038	0.153	0.031	0	0.028	0	0	0.209	0.001	0.000	0.000	0.190
GSM1423999_Treat	0	0.173	0	0.022	0	0.098	0.048	0.068	0.003	0.043	0.049	0.019	0.006	0.303	0.046	0	0	0	0.121	0	0.001	0.000	0.000	0.208
GSM1424000_Treat	0	0.205	0	0.034	0.15	0	0	0.036	0	0.051	0.038	0.039	0.027	0.297	0.003	0	0	0	0	0.097	0.023	0.000	0.000	0.145
GSM1424001_Treat	0.018	0.034	0	0	0.021	0.127	0	0	0.105	0.112	0	0.037	0.083	0.156	0	0.221	0	0.018	0.069	0	0	0.000	0.000	0.132
GSM1424002_Treat	0.109	0.035	0	0	0.155	0.042	0.025	0	0	0.052	0.067	0.002	0.028	0	0.054	0.291	0.012	0	0	0.066	0.046	0.016	0.000	0.197
GSM1424003_Treat	0	0.072	0	0	0.182	0	0	0	0.017	0.028	0.067	0.014	0.317	0.055	0.013	0.076	0	0	0.008	0	0	0.153	0.000	0.486
GSM1424004_Treat	0.068	0	0	0	0.106	0	0.042	0	0.024	0.096	0.041	0.005	0.066	0.13	0.057	0.149	0	0	0.179	0	0	0.037	0.000	0.201
GSM1424005_Treat	0.05	0.125	0.042	0.099	0.166	0	0.096	0	0	0.049	0	0.053	0.12	0.027	0	0	0	0	0.172	0	0	0.000	0.000	0.055
GSM1424006_Treat	0.015	0.005	0.015	0	0.107	0.065	0.018	0	0.047	0	0.241	0	0.11	0.123	0	0	0	0.004	0	0.075	0	0.174	0.000	0.199
GSM1424007_Treat	0.09	0.009	0	0	0.065	0.05	0.019	0	0	0	0.154	0	0.216	0.061	0.04	0.074	0	0	0.039	0	0.01	0.174	0.000	0.407
GSM1424008_Treat	0.165	0	0	0	0.01	0.181	0	0	0	0.008	0.003	0.114	0.042	0.064	0.042	0.057	0.016	0	0	0.14	0.069	0.087	0.000	0.279

Input Sample	B cells naive	B cells memory	Plasma cells	T cells CD8	T cells CD4 naive	T cells CD4 memory resting	T cells CD4 memory activated	T cells follicular helper	T cells regulatory (Tregs)	T cells gamma delta	NK cells resting	NK cells activated	Monocytes	Macrophages M0	Macrophages M1	Macrophages M2	Dendritic cells resting	Dendritic cells activated	Mast cells resting	Mast cells activated	Eosinophils	Neutrophils	P-value	Correlation
GSM1424009_Treat	0	0.102	0	0	0	0.065	0.037	0	0	0	0	0.008	0.302	0.014	0.045	0.216	0.011	0	0.126	0	0	0.074	0.000	0.411
GSM1424010_Treat	0	0.244	0.039	0.342	0	0	0.012	0.016	0.064	0.018	0	0.158	0	0.001	0	0	0	0	0	0.027	0.077	0.000	0.000	0.184
GSM1424011_Treat	0.058	0.054	0	0.051	0.037	0	0	0	0.072	0.126	0	0.007	0.151	0.115	0	0.151	0	0	0.155	0	0.023	0.000	0.000	0.146
GSM1424012_Treat	0.079	0	0	0.176	0	0	0	0.011	0.136	0	0	0.202	0	0.281	0	0	0.045	0	0	0.07	0	0.000	0.000	0.013
GSM1424013_Treat	0	0.095	0	0.086	0	0.159	0.055	0	0.007	0	0.01	0.028	0.059	0	0.012	0.239	0.06	0	0.124	0	0.042	0.022	0.000	0.158
GSM1424014_Treat	0.032	0.031	0	0	0.053	0.159	0	0	0	0.049	0.032	0.06	0.093	0.05	0	0.237	0	0	0.134	0	0	0.069	0.000	0.220
GSM1424015_Treat	0	0.151	0	0.064	0.099	0	0	0	0.048	0.043	0.043	0.009	0.032	0.244	0	0.095	0.007	0	0.11	0	0.054	0.000	0.000	0.203
GSM1424016_Treat	0.138	0.058	0	0	0	0.053	0.063	0	0	0.032	0.092	0.046	0	0.225	0.1	0	0	0	0.173	0	0.021	0.000	0.000	0.131
GSM1424017_Treat	0.007	0.073	0	0.034	0	0.182	0.007	0	0.024	0	0.081	0	0.049	0	0.033	0.317	0.066	0	0	0.085	0.032	0.010	0.000	0.243
GSM1424018_Treat	0.244	0	0	0	0.114	0.146	0	0	0	0.092	0.097	0	0	0.19	0	0	0.002	0	0	0.114	0	0.000	0.000	0.104
GSM1424019_Treat	0.255	0	0	0	0.051	0.115	0.017	0	0	0.079	0.06	0	0	0.236	0.003	0	0	0	0.185	0	0	0.000	0.000	0.041
GSM1424020_Treat	0.065	0.092	0	0.109	0.034	0.074	0.003	0	0	0	0.164	0	0.031	0.188	0.068	0	0	0	0.171	0	0	0.000	0.000	0.086
GSM1424021_Treat	0.058	0	0	0	0.009	0.163	0.103	0	0	0.038	0.221	0	0	0.229	0.053	0	0	0	0.127	0	0	0.000	0.000	0.222
GSM1424022_Treat	0.136	0	0	0	0.039	0.077	0.198	0.003	0	0	0	0.023	0	0.266	0.026	0	0.034	0	0.173	0	0.024	0.000	0.000	0.060
GSM1424023_Treat	0.131	0	0	0	0	0.173	0	0.045	0	0.198	0	0.088	0	0.218	0.009	0	0.075	0	0.008	0.047	0.009	0.000	0.000	0.090
GSM1424024_Treat	0	0.083	0	0.026	0.24	0.042	0	0.074	0	0.267	0	0.02	0	0.083	0	0.033	0	0	0.087	0	0.045	0.000	0.000	0.206
GSM1424025_Treat	0.163	0	0	0.018	0.009	0.087	0.016	0	0.008	0	0.113	0	0.042	0.2	0.021	0.158	0	0	0.142	0	0.023	0.000	0.000	0.205
GSM1424026_Treat	0.122	0	0	0	0.111	0.042	0	0	0	0.091	0	0.072	0.026	0.084	0.014	0.138	0.053	0.009	0.206	0	0.033	0.000	0.000	0.099
GSM1424027_Treat	0.138	0	0	0	0.095	0	0.089	0	0.093	0.096	0.089	0	0.036	0.261	0	0	0	0.017	0.085	0	0	0.000	0.000	0.126
GSM1424028_Treat	0.039	0	0	0	0.197	0	0.033	0	0	0.225	0	0.027	0.095	0.105	0.022	0.049	0.089	0	0	0.025	0.005	0.089	0.000	0.224
GSM1424029_Treat	0.042	0	0	0.018	0.103	0	0.119	0	0.009	0.017	0.07	0	0.129	0	0	0.269	0	0	0.152	0	0	0.072	0.000	0.220
GSM1424030_Treat	0.065	0.018	0	0	0.261	0	0.037	0	0	0.044	0.124	0	0.021	0.226	0.073	0	0	0	0.113	0	0	0.017	0.000	0.059
GSM1424031_Treat	0	0.125	0	0.151	0.067	0.067	0.135	0	0.009	0	0.249	0	0	0.143	0	0	0	0	0.054	0	0	0.000	0.000	0.086
GSM1424032_Treat	0.143	0	0	0	0.023	0.29	0	0	0	0.069	0.07	0.024	0	0.053	0.013	0.118	0.025	0	0.173	0	0	0.000	0.000	0.069
GSM1424033_Treat	0.014	0.08	0	0	0.153	0	0.05	0	0	0.082	0.121	0	0.081	0.022	0.033	0.027	0.001	0	0	0.184	0.03	0.121	0.000	0.265
GSM1424034_Treat	0.052	0.047	0	0	0.044	0.304	0.03	0	0	0.001	0.14	0	0.066	0.208	0	0	0.026	0	0.081	0	0	0.000	0.000	0.052
GSM1424035_Treat	0	0.136	0.006	0	0.173	0	0	0	0	0.041	0.113	0	0.015	0.181	0	0.031	0.025	0.002	0.145	0	0	0.134	0.000	0.222
GSM1424036_Treat	0	0.108	0	0	0.116	0.027	0.094	0	0	0.05	0.198	0	0.107	0.018	0.03	0.021	0.02	0	0	0.032	0.024	0.154	0.000	0.191
GSM1424037_Treat	0	0.261	0	0	0	0.141	0	0.01	0	0	0.08	0.045	0.047	0.246	0.028	0	0.025	0	0.118	0	0	0.000	0.000	0.119
GSM1424038_Treat	0.235	0	0.014	0.115	0.163	0	0.038	0	0	0.122	0	0	0	0.257	0	0	0	0	0.056	0	0	0.000	0.000	0.138
GSM1424039_Treat	0.022	0.093	0	0	0.24	0	0	0	0.07	0.145	0.06	0	0	0.105	0	0.148	0	0	0.117	0	0	0.000	0.000	0.124
GSM1424040_Treat	0.044	0.054	0	0	0.225	0	0.001	0	0	0.107	0	0.121	0	0.272	0	0	0	0	0.176	0	0	0.000	0.000	0.046
GSM1424041_Treat	0.175	0	0	0	0.028	0.089	0.047	0	0	0	0.08	0	0.075	0.16	0.013	0	0	0	0.092	0	0	0.243	0.000	0.243
GSM1424042_Treat	0.157	0	0	0	0	0.237	0	0.038	0	0	0.176	0.048	0.011	0.201	0	0	0	0	0.119	0	0.013	0.000	0.000	0.091
GSM1424043_Treat	0.224	0	0	0	0.135	0.315	0	0	0.005	0	0.122	0.04	0	0.083	0	0	0	0	0.036	0	0.041	0.000	0.000	0.047
GSM1424044_Treat	0	0.152	0	0	0.17	0.087	0.087	0	0	0	0.214	0	0	0.237	0	0	0.012	0	0.032	0.009	0	0.000	0.000	0.026
GSM1424045_Treat	0.307	0	0	0	0.099	0.196	0	0.047	0	0.014	0.105	0	0	0.148	0	0	0	0	0	0.084	0	0.000	0.000	0.055
GSM1424046_Treat	0.107	0	0	0.051	0.043	0	0	0.012	0.118	0	0.029	0.092	0.06	0.124	0.002	0	0	0	0.12	0	0	0.241	0.000	0.266
GSM1424047_Treat	0.102	0.014	0	0	0.255	0	0	0	0.04	0	0.089	0.027	0.032	0.179	0.012	0.001	0	0	0.175	0	0.075	0.000	0.000	0.124
GSM1424048_Treat	0.177	0.046	0	0	0.077	0.135	0	0	0.026	0	0.159	0	0.033	0.067	0	0.072	0	0	0.01	0.028	0.049	0.120	0.000	0.154
GSM1424049_Treat	0.035	0.106	0	0.072	0	0.051	0.018	0.025	0	0	0.047	0.067	0	0.186	0.022	0.031	0.163	0	0.177	0	0	0.000	0.000	0.040
GSM1424050_Treat	0.097	0	0	0.039	0	0.303	0.067	0	0.022	0	0.075	0.069	0.026	0.115	0.017	0	0.063	0	0	0	0.108	0.000	0.000	0.062
GSM1424051_Treat	0.062	0.102	0	0	0.012	0.203	0	0	0	0	0.177	0.038	0	0.26	0.024	0	0	0	0.122	0	0	0.000	0.000	0.130
GSM1424052_Treat	0.205	0	0	0	0.167	0.086	0.087	0	0	0.078	0.079	0.01	0	0.149	0.037	0	0	0	0.094	0	0.009	0.000	0.000	0.118
GSM1424053_Treat	0	0.074	0.028	0	0.038	0.129	0.058	0	0.038	0.051	0.054	0	0	0.06	0.01	0.237	0.046	0.027	0.135	0	0	0.018	0.000	0.108
GSM1424054_Treat	0	0.216	0.004	0.184	0.006	0.039	0.109	0	0	0.005	0.144	0	0	0.139	0.048	0.024	0	0	0.063	0	0.02	0.000	0.000	0.198
GSM1424055_Treat	0.195	0	0	0	0.113	0.109	0.089	0.009	0	0.143	0.065	0	0.059	0.155	0	0	0	0	0.063	0	0	0.000	0.000	0.163
GSM1424056_Treat	0.064	0.061	0	0.07	0	0	0.026	0	0.054	0.044	0	0	0.067	0.125	0.034	0.158	0.005	0	0.175	0	0.051	0.066	0.000	0.167
GSM1424057_Treat	0	0	0.065	0.075	0.02	0	0.063	0	0.054	0	0.023	0.017	0.201	0.071	0.022	0	0.078	0	0	0.242	0	0.070	0.000	0.141
GSM1424058_Treat	0	0.278	0.05	0	0.184	0	0	0	0	0	0.094	0.004	0	0.223	0.009	0.057	0.049	0.002	0.05	0	0	0.000	0.000	0.146
GSM1424059_Treat	0.163	0	0	0.245	0	0	0	0.059	0	0	0	0	0.057	0	0	0.212	0.076	0.02	0	0.044	0.125	0.000	0.000	0.132
GSM1424060_Treat	0.024	0.022	0	0.028	0	0.101	0.077	0	0	0.107	0	0	0.043	0	0.045	0.338	0.058	0	0.052	0	0.037	0.067	0.000	0.355
GSM1424061_Treat	0.041	0	0	0	0.177	0	0.055	0	0.168	0.143	0	0.028	0.175	0.11	0	0	0	0	0.104	0	0	0.000	0.000	0.218
GSM1424062_Treat	0.014	0.106	0	0	0.126	0.089	0.044	0.101	0	0.146	0	0.012	0	0.009	0	0.147	0.018	0	0.154	0	0.033	0.000	0.000	0.049
GSM1424063_Treat	0.154	0	0	0	0.179	0	0	0	0.028	0	0.032	0.024	0.156	0	0.001	0.219	0	0	0.107	0	0	0.100	0.000	0.226
GSM1424064_Treat	0	0.09	0.024	0.254	0	0	0	0.111	0	0.011	0	0.119	0	0.071	0.025	0.052	0.014	0	0.229	0	0.001	0.000	0.000	0.088
GSM1424065_Treat	0	0.09	0.154	0	0.047	0.164	0.055	0	0	0	0	0.117	0	0.072	0	0	0	0.015	0.287	0	0	0.000	0.000	-0.076
GSM1424066_Treat	0	0.064	0.007	0.138	0	0.033	0.08	0	0	0.084	0	0.056	0.126	0.041	0	0.059	0.03	0	0.224	0	0	0.056	0.000	0.153
GSM1424067_Treat	0.16	0	0	0	0	0.235	0.074	0	0.001	0	0.17	0	0.092	0	0	0	0.045	0	0.183	0	0.041	0.000	0.000	0.014
GSM1424068_Treat	0	0.156	0	0.238	0	0.067	0.155	0	0	0	0	0.052	0	0.055	0.04	0.099	0.019	0	0.119	0	0	0.000	0.000	0.226
GSM1424069_Treat	0	0.189	0	0.281	0.023	0	0.217	0	0	0	0.001	0	0.065	0	0	0	0.062	0	0.162	0	0	0.000	0.000	0.073
GSM1424070_Treat	0	0.173	0	0.272	0.038	0	0.075	0	0	0.044	0	0.081	0	0.074	0.051	0.055	0.047	0	0.088	0	0	0.000	0.000	0.081
GSM1424071_Treat	0.025	0	0.005	0.074	0.008	0.137	0.05	0	0	0	0.109	0	0.258	0	0.011	0.104	0	0	0.053	0	0	0.167	0.000	0.262
GSM1424072_Treat	0.044	0	0	0	0.109	0.072	0	0.232	0	0	0.091	0.017	0	0.187	0	0	0.127	0	0.122	0	0	0.000	0.000	-0.040
GSM1424073_Treat	0	0.012	0	0.209	0.093	0.12	0.093	0.042	0	0	0	0.084	0.005	0.112	0	0.032	0	0	0.198	0	0	0.000	0.000	-0.006
GSM1424074_Treat	0	0.067	0	0.286	0	0.135	0.037	0.046	0.008	0	0	0.045	0	0.074	0	0.086	0	0	0.218	0	0	0.000	0.000	-0.016
GSM1424075_Treat	0	0.054	0.133	0	0.285	0	0.025	0.081	0	0	0	0.203	0	0.092	0	0	0.031	0	0.032	0	0.065	0.000	0.000	0.064
GSM1424076_Treat	0	0.244	0	0	0.145	0.05	0	0	0	0	0.064	0.014	0	0	0	0.256	0.017	0	0.139	0	0.071	0.000	0.000	0.079
GSM1424077_Treat	0.108	0.057	0	0.057	0.051	0.009	0.115	0	0	0.14	0.048	0	0.016	0	0	0.166	0.036	0.054	0.132	0	0	0.012	0.000	0.165
GSM1424078_Treat	0	0.067	0.127	0.322	0	0	0	0.149	0	0	0	0.151	0	0.056	0	0	0	0	0.04	0	0.088	0.000	0.000	0.061
GSM1424079_Treat	0	0.11	0.05	0.171	0	0	0	0	0.091	0	0.035	0	0.026	0	0	0.097	0	0	0	0.188	0	0.231	0.000	0.119
GSM1424080_Treat	0.057	0	0	0.028	0	0.086	0.003	0.021	0	0	0	0.027	0.189	0	0	0.28	0.005	0.017	0.155	0	0	0.131	0.000	0.335
GSM1424081_Treat	0.049	0.068	0	0	0.043	0.244	0	0.069	0	0	0.137	0	0	0.004	0	0.134	0	0.085	0	0.122	0	0.045	0.000	0.090
GSM1424082_Treat	0.172	0	0	0	0.093	0.138	0.05	0	0	0	0.112	0	0.039	0.04	0.017	0.106	0.017	0.03	0.114	0	0	0.071	0.000	0.094
GSM1424083_Treat	0	0.199	0	0.099	0	0.241	0	0	0	0	0.09	0.041	0	0.122	0	0	0	0	0.121	0	0.087	0.000	0.000	0.003
GSM1424084_Treat	0	0.195	0.024	0	0.203	0.008	0.005	0	0	0.034	0.055	0	0.051	0.023	0.025	0.17	0.02	0	0.098	0	0	0.089	0.000	0.149
GSM1424085_Treat	0	0.154	0	0	0	0.33	0	0	0.032	0	0.108	0	0.024	0.097	0	0	0.006	0.108	0	0.102	0	0.038	0.000	0.016
GSM1424086_Treat	0	0.199	0	0.155	0	0.027	0.09	0.069	0	0	0.027	0.042	0.001	0.047	0	0	0.02	0	0.308	0	0.016	0.000	0.000	0.046
GSM1424087_Treat	0.006	0.074	0	0.016	0	0.118	0.078	0.026	0	0.032	0.068	0	0.014	0	0.006	0.268	0.124	0	0.16	0	0.01	0.001	0.000	0.273
GSM1424088_Treat	0	0.056	0	0	0.053	0	0	0	0	0.039	0.063	0	0.327	0.01	0.023	0.238	0	0	0.102	0	0	0.090	0.000	0.324
GSM1424089_Treat	0.111	0	0	0.041	0	0.079	0	0	0.005	0	0.219	0	0.228	0.049	0.034	0	0.061	0	0	0.044	0.012	0.117	0.000	0.185
